# Highly Asynchronous and Asymmetric Cleavage Divisions Accompany Early Transcriptional Activity in Pre-Blastula Medaka Embryos

**DOI:** 10.1371/journal.pone.0021741

**Published:** 2011-07-07

**Authors:** Michael Kraeussling, Toni Ulrich Wagner, Manfred Schartl

**Affiliations:** Institute of Science and Technology Austria, Austria

## Abstract

In the initial phase of development of fish embryos, a prominent and critical event is the midblastula transition (MBT). Before MBT cell cycle is rapid, highly synchronous and zygotic gene transcription is turned off. Only during MBT the cell cycle desynchronizes and transcription is activated. Multiple mechanisms, primarily the nucleocytoplasmic ratio, are supposed to control MBT activation. Unexpectedly, we find in the small teleost fish medaka (*Oryzias latipes*) that at very early stages, well before midblastula, cell division becomes asynchronous and cell volumes diverge. Furthermore, zygotic transcription is extensively activated already after the 64-cell stage. Thus, at least in medaka, the transition from maternal to zygotic transcription is uncoupled from the midblastula stage and not solely controlled by the nucleocytoplasmic ratio.

## Introduction

The early development of many animals apparently follows a common scheme: it is characterized by a rapid and synchronous sequence of cell divisions [1] [2]. During this period, the cleavage-phase, the single cell of the fertilized egg is divided into a large number of blastomers. This bulk of cells will finally form the blastula. While the embryo reaches the blastula stage, the cells pass through an important time point of early development, which is called midblastula transition (MBT). This process changes both characteristics and behavior of the cells. Before MBT, the cell cycle is highly synchronous. It is rapid because the cells lack both G1- and G2-phases [3] and there is no zygotic gene expression [1] [4] [5] [6]. The cell cycle elongates and desynchronizes only after the cells have passed through the MBT. Cells gain motility and start to express the zygotic genome while maternal mRNAs are degraded [7].

The time points of activation of the midblastula transition vary between different species. MBT starts early in *Drosophila* at stage 4 (embryonic division cycle 11) [8], in Xenopus at stage 8 (cycle 12) [1] [3] [9] [10] , in Zebrafish at stage 10 (cycle 10) [2] and earlier work identified the beginning of MBT for medaka fish at around stage 11 (cycle 11–12) [6].

At present, four models exist that try to explain MBT regulation: the maternal clock [11] [12], transcriptional abortion [5] [13], chromatin regulation [14] [15], and the nucleo-cytoplasmic ratio.

Among these, the regulation by a nucleo-cytoplasmic ratio is the oldest and best established model [1] [2] [9]. It proposes that suppressor molecules are present in the cytoplasm of the unfertilized egg and block several events like activation of zygotic gene expression [1]. During the first cell divisions, these hypothetical factors will be titrated out by the increasing number of nuclei relative to the constant total volume of cytoplasm. As soon as the concentration of repressing factors drops below a certain threshold, they will lose their repressing potential and MBT will start.

This hypothesis has been supported by functional studies using nuclear transplantations and experimental manipulation of the cytoplasmic volume. Such experiments resulted either in a delayed or a premature beginning of the MBT [2] [3]. Data from haploid [5] or tetraploid [2] [5] animals strengthened these observations. Addition of extra DNA also led to an earlier start of the MBT [16] [17] [18].

Molecular, cellular and embryonic processes at early stages before MBT are neither well characterized nor fully understood for teleosts in general and medaka (*Oryzias latipes*) in particular. The medaka is a laboratory fish model of growing importance. It is comparable to zebrafish and also holds many features that legitimate it as a useful complementary model system [19]. For developmental studies, both model systems are of great interest since fertilization of eggs and embryonic development are external and embryos are totally transparent throughout the complete embryonic development.

However, medaka may be more expedient than zebrafish for a couple of approaches that deal with the very early embryogenesis because of its slower embryogenesis. Zebrafish hatch after 2–3 days post fertilization, whereas medaka embryos develop much slower and do not hatch until day 7.

While a lot of research aiming at MBT in fish in general has been performed using zebrafish [2] only one recent study concentrated on medaka [6]. The fact that the MBT of both species starts at different time points gives a first hint that data on zebrafish MBT might not be representative for all fish species and that a closer look on medaka embryos is justified.

Unexpectedly, we find that a large number of medaka embryos show highly asymmetric cell divisions as early as from the second cell division onwards. This asymmetry is directly reflected in different cell sizes and cytoplasmic volumes, but surprisingly without negative effects on normal development. Medaka embryos also lose cell-cycle synchrony already at cleavage 5, leading to transition from synchronous to metasynchronous cell division. Most surprisingly, we observed strong RNA polymerase II phosphorylation down from the 64-cell stage and the subsequent initiation of mRNA transcription. This might be connected to regulatory processes that prepare embryos for the MBT or even mark the beginning of MBT itself at a much earlier time point than has been reported previously.

## Results

### Asynchronous cell divisions in early embryos

The cell cycle during cleavage stages is generally described as being short and highly synchronous. Lengthening of the cell cycle and upcoming asynchrony is taken as a sign for the beginning MBT [1]. For Zebrafish, this event has been mapped to cycle 10 [2]. Time-lapse investigations in our laboratory on medaka fish provided preliminary evidence that embryos from this species lose cell division synchrony several stages before MBT occurs in zebrafish, and establish a cell division behavior that is called “metasynchronous cell division” (Movie S1).

**Figure 1:**
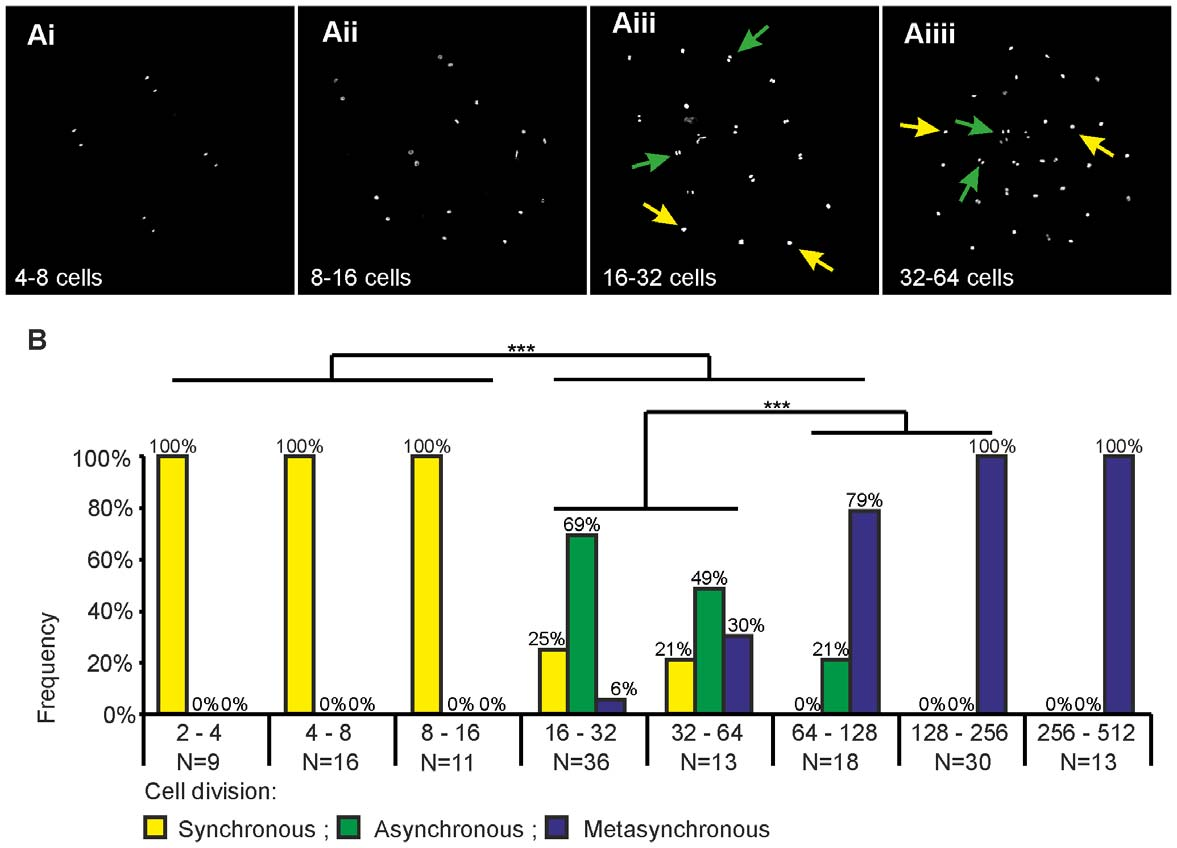
Desynchronization of cell cycle in early medaka embryos. Progression of cell cycle desynchronization in early medaka embryos before MBT. (A) Image shows staining for DNA at 4 different cell divisions (4–8 cells, 8–16 cells, 16–32 cells, 32–64cells). (Ai–Aii) Synchronous cell division at cycle 4 to 8 cells and at cycle 8 to 16 cells. (Aiii–Aiiii) Asynchronous cell division at cycle 16 to 32 cells and at cycle 32 to 64 cells. (Aiii) Asynchronous cells at cleavage from 16 cells to 32 cells at random positions in the embryo; nuclei are at interphase (yellow arrows) or at late mitotic phase (green arrows). (Aiiii) Asynchronous cleavage from 32 to 64 cells, cells in the center are in late mitotic phase (green arrows), most cells at the rim are at interphase (yellow arrows). (B) Progression from synchronous to asynchronous and from asynchronous to metasynchronous cell division between cycle 2 (2–4 cells) and cycle 9 (256–512 cells). Embryos that divided synchronously are represented by yellow bars, embryos that divided asynchronously by green bars and metasynchronously dividing embryos by blue bars. Note that more synchronous dividing embryos are found during the first three cell divisions (2–4 cells, 4–8 cells , 8–16 cells) than during the second three cell divisions (16–32 cells, 32–64 cells, 64–128 cells) (Chi-test with p<0.001; for this, metasynchronous divisions were also counted as asynchronous divisions). Later, the number of embryos showing a random asynchronous division pattern drops and more and more embryos show a clear metasynchronous cell division. By the division from 64–128 cells, the majority of the division pattern has changed from asynchronous to metasynchronous (p<0.001).

In order to investigate this phenomenon further, confocal imaging was used to visualize differences in cell synchrony at defined stages. We found that cell division is synchronous in embryos until cell division 4, when embryos progress from 8 to 16 cells. First indications for an upcoming asynchrony appear at cell division 5, 16 to 32 cells, when a temporal spacing of the cell cycle between single cells can be detected (Fig. 1A).

During the following division cycles, cell division synchrony in embryos is not lost completely. Rather, it is replaced by a specific division pattern that is called “metasynchrony”, which is defined by cell division progressing in waves that start in the embryos' center and spread out to their periphery. In early medaka embryos, cells are usually arranged in an elongated or rectangular manner until the 16-cell stage and in a roundish, disc-like manner from the 32-cell stage on until gastrulation. Starting at cycle 6 (32 to 64 cells), when embryos have a sufficient number of cells to form well-defined central and peripheral regions, more and more embryos (5/13) are found in which a temporal spacing of mitosis initiation between central and peripheral cells can be identified (Fig. 1A, B).

This temporal spacing of mitosis initiation increases during the following divisions. At the latest with division 8 (128 to 256 cells) it reaches an extent at which all central cells have already re-entered interphase while peripheral cells are still in late anaphase or telophase (Fig.1B, Fig. S1).

**Figure 2:**
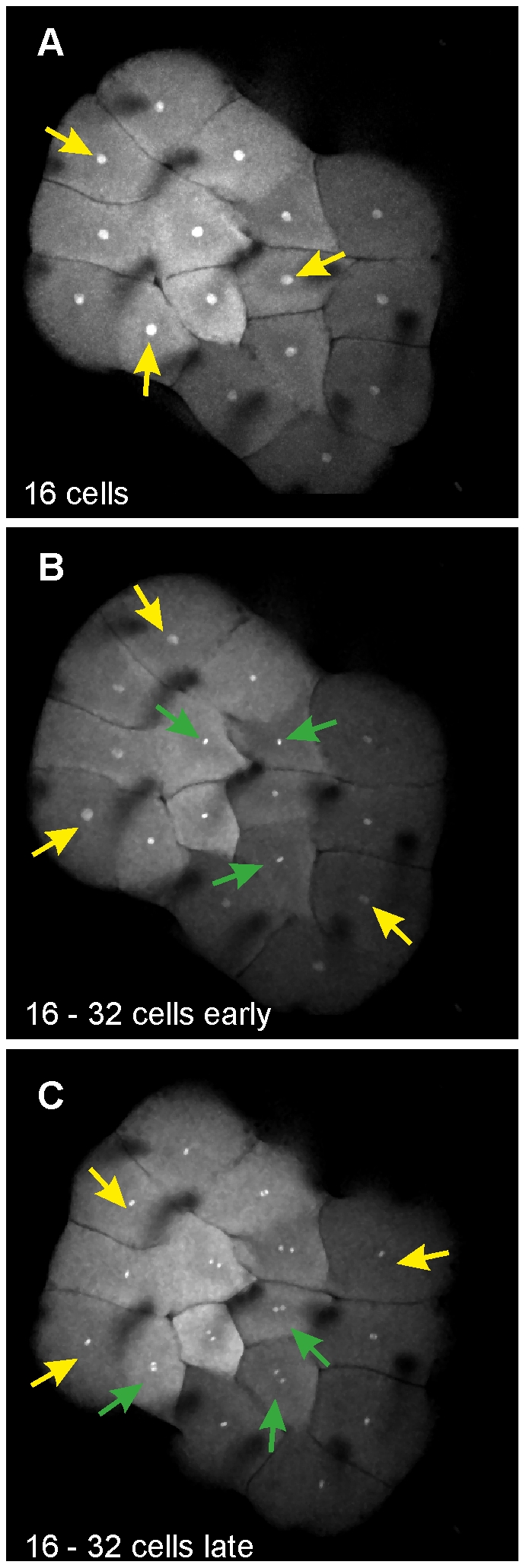
Asynchronous cell division appears in living embryos at cycle 5. Maximum intensity collapses of an embryo at 3 different time points of cycle 5 (16 to 32 cells) during time laps observations. (A) Nuclei of interphase cells are large due to the decondensed chromatin. (B) The nuclei in some cells are shrinking when their chromatin starts to condensate (green arrows), while the chromatin of other cells remains decondensed (yellow arrows). (C) Some cells are at ana-/ or telophase (green arrows), while remaining cells only have started to condensate their chromatin (yellow arrows).

These findings were confirmed by time-lapse observations of embryos that were fluorescently tagged by injection of mRNA for an eGFP labeled Histone2B protein (H2B-eGFP) and imaged throughout the cleavage phase. These embryos showed highly synchronous cell divisions until the 16-cell stage and an emerging minor temporal spacing during the division from 16 cells to 32 cells (Fig. 2). During the following divisions, this temporal spacing again increased until cell division initiation could be clearly detected first in centrally located cells and later in peripheral cells. For example, karyokinesis at division 7 (64 to 128 cells) has finished in central cells while peripheral cells are still at late anaphase or telophase (Fig. S2, Movie S2).

Tracking the size (as a determined area) of a single cell's nucleus and its daughter cells from a mid 8-cell stage to the late 64-cell stage at constant time intervals for 45 consecutive measurement points illustrates the relationship between the metasynchronous cell cycle and the position of a cell within the embryo. Nuclei have their largest area during interphase and this value decreases in preparation for the next mitosis. The nuclear signal reaches its smallest area at the metaphase when daughter chromosomes are separated. Comparing the 4 displayed daughter cells at the passage from 32 to 64 cells, the most central cell has condensed its chromatin one measurement point earlier than the other 3 cells. Later, the daughter cells of this early cell have entered cell division two measurement points before the others (Fig. S3).

Furthermore, some of the embryos that were observed during the time lapse experiments, divided asymmetrically from the 2-cell to the 4-cell stage. In these embryos cell division also de-synchronized at the cycle from 16 to 32 cells, but at the following stages not each one of these embryos did develop the pattern typical for the symmetrically dividing embryos described above, where central cells cycled first and peripheral cells later (Fig. S4). In the given example, cells first started division at one pole of the embryo and cells on the opposite pole followed later (Fig. S5, Movie S3).

### Asymmetric cleavage at the 2-cell stage does not affect embryo development

**Figure 3:**
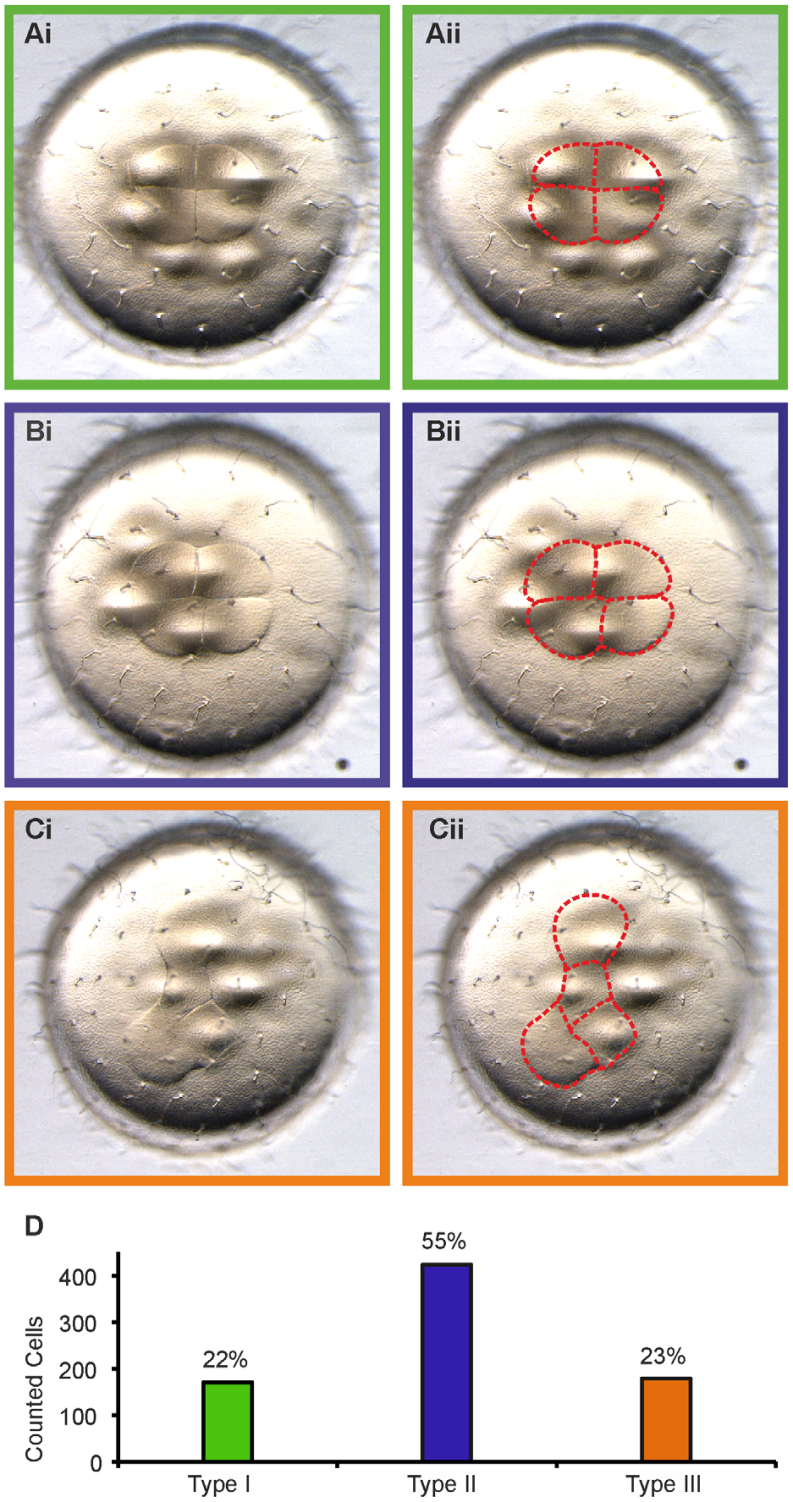
Asymmetric cell divisions at cycle 2. (Ai, Bi, Ci) Characterization of three types of medaka embryos at the 4-cell stage with regard to the grade of axial symmetry of the cell cleavages. (Ai) Type I embryo showing highly symmetric cleavages and right-angled cells. (Aii) Cell boundaries were highlighted to help visualization. (Bi; Bii) Type II embryo showing slight but clearly detectable impairment of axial symmetry, while still having right-angled cell shapes at the interception point. (Ci; Cii) Type III embryos lacking axial symmetry. (D) Frequency of the three embryo types at 4-cell stage medaka embryos among 774 eggs. Type I occurred with a frequency of 22% (171/774) of total embryos (green bar), type III embryos with 23% (179/ 774) (orange bar). Type II embryos represent the largest group of the three types with 55% (424/774) of all embryos (blue bar).

Time-lapse observations of injected medaka embryos showed the occurrence of highly asymmetric cleavages from the 2-cell to the 4-cell stage. For a detailed analysis the cleavage furrows and the cell-arrangements of medaka embryos at the 4-cell stage were investigated and consequently three distinct classes of embryos were defined. They were designated type I, type II and type III (Fig. 3). Embryos with almost perfect 90° cleavage furrows in the center of the four cells and symmetry along the X and Y axes were classified as type I embryos (Fig. 3Ai, Aii). Generally, these embryos have four homomorphic cells, yet we occasionally observed that two of the four cells were seemingly smaller than the others. Type II embryos still feature 90° cleavage furrows but have lost axial symmetry (Fig. 3Bi, Bii). The interception point of the cleavage furrows is located at the center of the four cells, but the furrows no longer build a cross-like structure. In a type I embryo, cells only have contact to their directly neighboring cells, but in a type II embryo two cells now also have contact to their opposing cell, forming an hourglass-like structure. Type III embryos include all the embryos that lack any symmetric cleavage furrow or clear organization of the cells or both (Fig. 3Ci, Cii; Fig. S6).

To investigate if asymmetric cleavages have a negative effect on embryonic development, the survival rate of the three different cleavage types was determined. Altogether, 774 embryos were classified according to their cleavage furrows: 171 belonged to type I (22%), 424 to type II (55%) and 179 to type III (23%) (Fig. 3D). These embryos were raised under standard conditions and monitored for early embryonic death. Surprisingly, among the 774 embryos, only one died before hatching and this one was scored as type II. The remaining embryos developed normally and hatched on time around day 7 post fertilization.

Time-lapse observations of developing embryos from the 4-cell stage up to the 1024-cell stage were performed to investigate possible effects of the cleavage type on development. Type I embryos developed just like the idealized medaka embryo as described by Iwamatsu [20]. They start with a typical clover leaf shape and stay symmetric until stage 10, 1024 cells, when the embryos reach the typical roundish disc of the early blastula stage (Fig. S7). Type II embryos differ only at early stages from Iwamatsús ideal embryo. At the 8-cell or 16-cell stage, embryos sometimes showed slightly shifted shapes as cells were not arranged in a symmetric manner. These shape differences were usually compensated until the 32-cell or 64-cell stage, respectively. Even type III embryos were able to establish the typical disc of the early blastula stage. However, in these embryos this process required more cell divisions and thus more time depending on the grade of deviation from the type I development (Fig. S8). In the given example, the embryo was not able to fully compensate before 1024 cells (Fig. S9).

### Unequal cell volumes at the 4-cell stage

**Figure 4:**
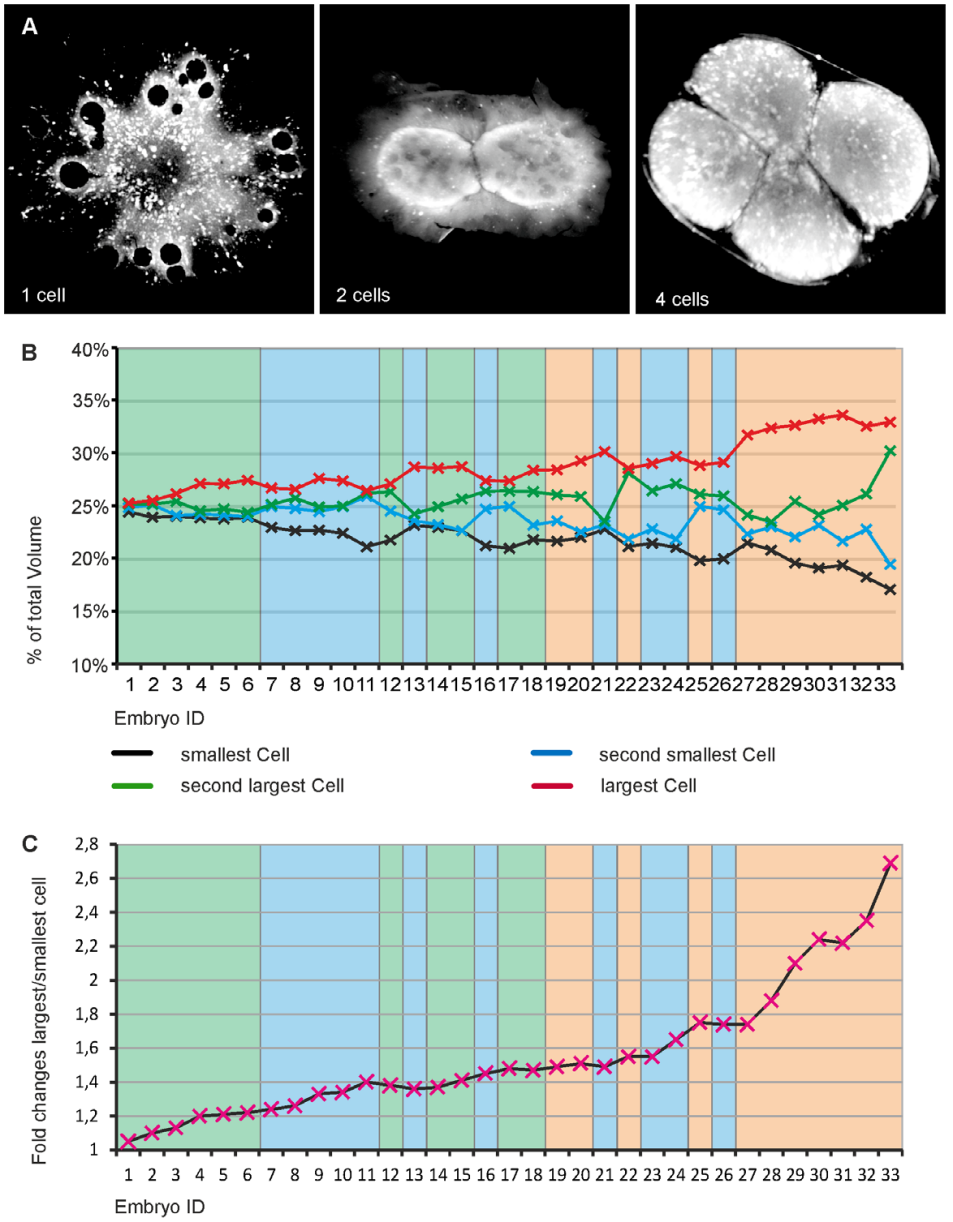
Cell volumes at the 4-cell stage. Variability of cell volumes at the 4-cell stage. (A) Maximum intensity z-projections of confocal stacks of medaka embryos at the 1-cell, 2-cell and 4-cell stage. Diffuse cellular boundaries during the 1-cell and 2-cell stages cannot be detected by software protocols. Cell membranes at the 4-cell stage are sharp and clearly separated between single cells and yolk. (B) Volumes from cells of different embryos. Graph displays the divergence of cellular volumes within individual embryos at 4-cell stage. X-axis shows individual embryos with increasing cell-volume differences to the right. (C) Relative factor differences between the largest and the smallest cell of individual embryos. (B and C) Background colors within the graphs indicate the embryo-type: type I embryos are represented by green, type II embryos by blue and type III embryos are represented by orange.

In order to measure cellular volumes, embryos were stained with CellMask DeepRed. However, this technique is not useful for measuring embryos before the 4-cell stage because the cellular boundaries to the cytoplasm are not apparent enough to clearly define the shape of the cell. Only at the 4-cell stage the cellular border has reached a level of clarity that allows the discrimination of single cells (Fig. 4A). Fluorescent staining and confocal imaging technique was chosen in order to monitor changes in all three dimensions (Fig. S10).

A total of 33 embryos were scanned (11 type I, 11 type II and 11 type III) (Fig. 4B, Table S1). Of these embryos showed only very few similar or equal cell volumes (5/33). Many consisted of three cells of similar volume and a relatively large or small fourth cell (20/33).

If cell volumes are examined with respect to the three embryo types, it appears that the level of asymmetry is reflected by the level of differing cell volumes. In particular, 9/11 of type I embryos were found within the 16 embryos with the most similar volumes between the largest and the smallest cell. In contrast to that, 11/11 of type III embryos were found within the 17 embryos with the most different volumes between the largest and the smallest cell (Fig.S11). However, there is no strict correlation since some type I embryos were found that differed more in cell volumes than type II embryos as well as some type II differed more than type III (Fig. S12). The fold changes between the largest and the smallest cell of each embryo were calculated (Fig. 4C, Table S1). The smallest observed fold change in an embryo was 1.05. Most embryos ranged within a fold difference between 1.3 and 1.5 (15/33), but values up to 2.69 were found as well. Type I embryos range from 1.05 to 1.47 times difference, type II from 1.24 to 1.74 and type III from 1.49 to 2.69 (Table S1, Fig. S13).

### RNA polymerase II phosphorylation and transcriptional activity in early Medaka

**Figure 5:**
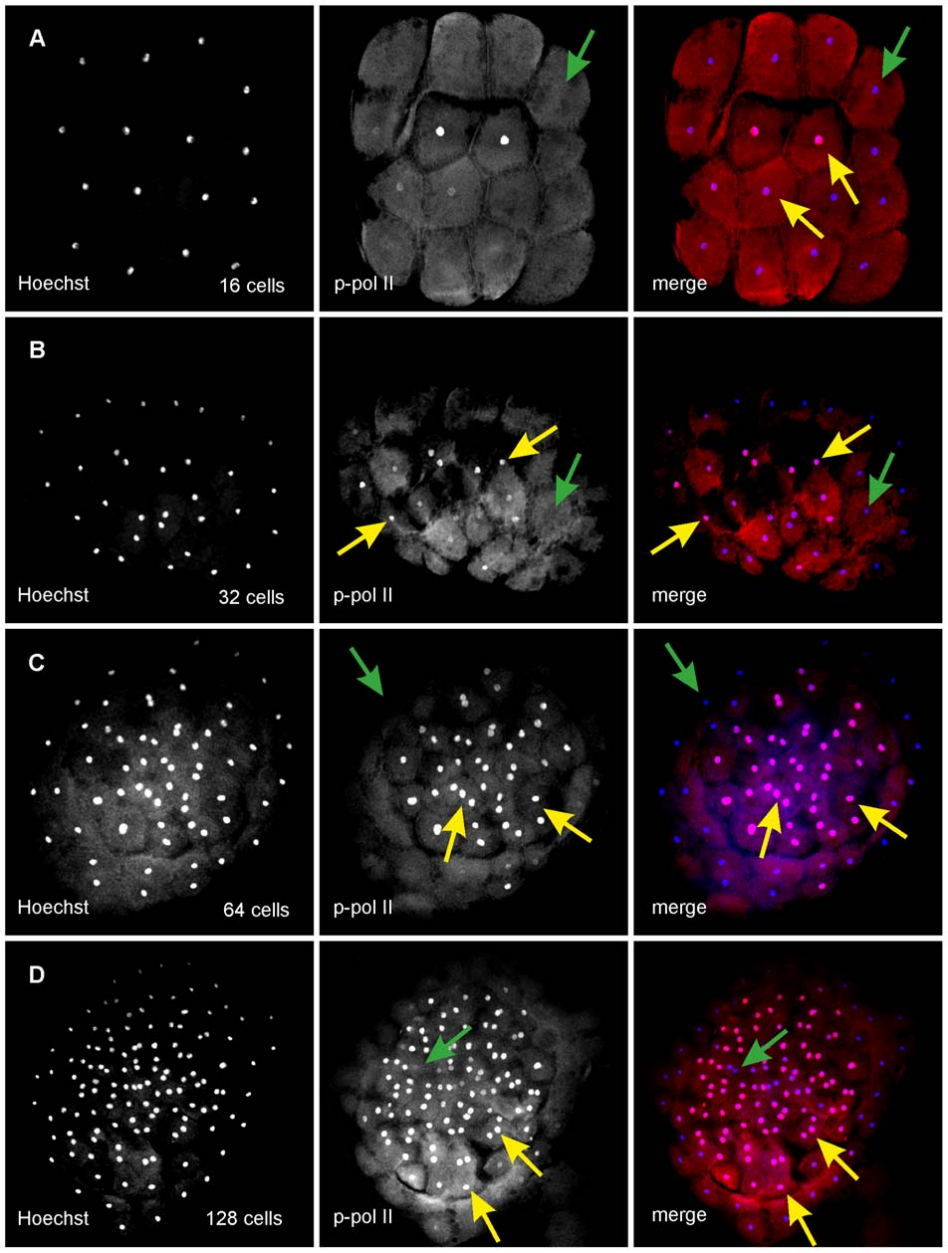
Polymerase II phosphorylation in embryos before MBT. Phosphorylation of mRNA polymerase II in medaka embryos at 8 to 16 cells (A), 32 cells (B), 64 cells (C) and 128 cells (D). (A) p-Pol II staining, first detected in cells during the late stages of mitosis between the 8-cell stage and the 16-cell stage. (B) p-Pol II signal in isolated cells at 16-cell stage. (C) 64-cell stage showing high levels of p-Pol II signal. Almost no phosphorylation is seen in peripheral cells, but ubiquitous signal in central cells. (D) 128-cell stage. Almost no p-Pol II positive nuclei were found in peripheral cells, most central nuclei stain positively.

To monitor the initiation of zygotic transcription, we started with the investigation of RNA polymerase II (RNAPII) phosphorylation. Unexpectedly, earliest phosphorylation was already detected in nuclei of a fraction of cells in embryos at the 16-cell stage (Fig. 5A). Positively stained nuclei remained rare during 16-cell and 32-cell stages with 17% of all embryonic cells showing positive staining at the 16-cell stage and 30% at the 32-cell stage respectively. A specific spatial pattern for this time points could not be identified (Fig. 5A, B).

By the 64-cell stage, the number of phospho-RNAPII positive cells increased to 73% (Fig S15). Cells that were not stained positive were exclusively located at the periphery and never at central positions (Fig. 5C).

At the 128-cell stage, the phosphorylation level was still high (68% of all cells). Peripheral cells were still negative for phospho-RNAPII, but negative cells or cells with very low levels of phosphorylation were now also found at more central positions of the embryo (Fig. 5D). Positive and negative cells were intermingled during the 256- and 512-cell stage (Fig. S14B) and peripheral cells showed polymerase II phosphorylation only after the 1024-cell stage (Fig. S14C).

**Figure 6:**
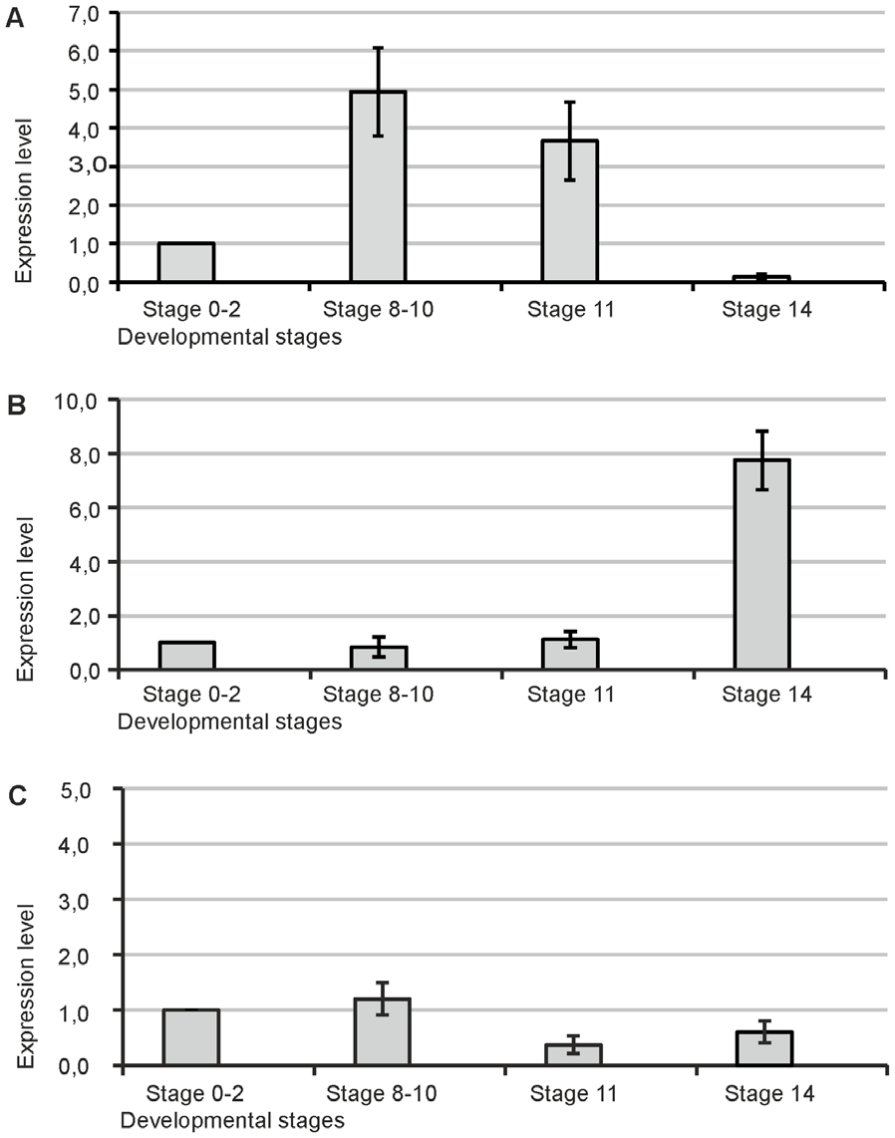
Quantitative gene expression analysis of Ccnb1, Rsp12 and PSMC1 during early embryogenesis. Expression levels of 3 candidate genes during 4 different time periods (Stages 0–2, Stages 8–10, Stage 11, Stage 14). (A) Expression of Ccnb1 is strong during stages 8–10 (p<0,0268) and stage 11 (p<0,0452). (B) Expression of Rsp12 shows induction at stage 11 and robust upregulation at stage 14 (p<0,0042). (C) Expression of PSMC1 is not upregulated. Expression levels at stages 0–2 were set as 1. (Significance was tested with the Student's t-test, error bars are standard deviations).

Transcriptional activity at these early stages was verified by RT-PCR. For this, a selection of target genes were investigated for transcriptional up-regulation at stages 0–2 (0–2 cells), 8–10 (64–1000 cells), 11 (early-late blastula), and stage 14 (pre-mid gastrula). Ccnb1, a member of the AB subfamily of cycline proteins that control the G2/M transition, showed strong up-regulation between stages 8 to 10 and 11. RPS12, which encodes for a member of the 40S ribosomal subunit, showed induction at stage 11 and robust induction at stage 14. PSMC1, a protease, (and four other genes, data not shown), showed no upregulation during the investigated stages (Fig. 6).

## Discussion

### Asynchronous and metasynchronous cell division starts between the 16 and 64 cells stages

In the classical studies on cell cycle duration in early vertebrate embryos data were obtained from time-lapse observations, which were based on standard light microscopy. We used confocal microscopy to detect earliest differences in cell cycle synchrony. With this technique, it was possible to detect first alterations and asynchrony at cycle 5 (from 16 to 32 cells) when the first cells became apparent that divided prior to the remaining cells. Asynchrony is a direct result of cell cycle lengthening at beginning MBT in *Drosophila*, *Xenopus* and zebrafish [2] [5] [13]. However, the loss of synchrony in medaka embryos at cycle 5 is not connected to a beginning MBT, but marks the beginning of a metasynchronous cell cycle.

The cell cycle in zebrafish embryos is rapid and synchronous from 2 to 128 cells. Metasynchronous cell division emerges at cycle 8 (from 128 to 256 cells) [21] and is well established at the early blastula stage at cycle 9 (from 256 to 512 cells) [2]. Although medaka embryos lose overall synchrony already at cycle 5, and start to develop a temporal spacing of mitosis initiation between central and peripheral cells right away. However, embryos at the 16-cell or 32-cell stage do not possess sufficient cells to form a distinct center and periphery or a distinct difference between both areas. This is probably the reason why it takes up to two or three additional cell divisions, until cycle 7 to 8, before embryos first displayed a cell cycle that occurs in clear waves - a typical feature of metasynchronous cell division.

Interestingly, this metasynchronous division pattern is distorted in embryos with asymmetric early cleavage furrows. Usually, embryos during cleavage phase show several levels of axial symmetry. At the 4-cell stage, cells are oriented in a clover leave like configuration, followed by a double row of 2×4 cells at the 8-cell stage, the 4×4 “chessboard” at the 16-cell stage and finally the upcoming roundish, disk-like arrangement at 32 cells and later stages. Embryos not showing this high level of symmetry are also more likely to be unable to establish a clear metasynchronous cell division, although they still establish a certain level of structured and organized division pattern. However, the regulatory mechanism behind this behavior remains unclear.

### Asymmetric cell cleavages and unequal cell volumes at the 4-cell stage

Asymmetric and unequal cell divisions occur in medaka already at the 4-cell stage. These asymmetric divisions produce divergent shapes of embryos instead of embryos consisting of cells that are even in shape and volume like a symmetric cell division would produce, and like it was described for medaka by Iwamatsu [20]. Our study demonstrates that only about one quarter of embryos follow the scheme of the idealized embryo with highly symmetric cell divisions. More or less strong deviations of the symmetric divisions represent the majority of possible cleavages and another quarter of embryos show extremely asymmetric cleavages. However, the deviation from symmetry has no influence on the further course of embryonic development. Unequal cell cleavages have already been reported for lower animals like leech [22], *Caenorhabditis elegans* [23] and sea urchin [24], but to our knowledge never before for a vertebrate.

MBT in general is regarded to be regulated by the nucleo-cytoplasmic ratio [1] [2] [9]. Consequently, unequal cell volumes, if differences are big enough, should cause alterations in MBT onset [3] [5]. Unexpectedly, as our study shows, already cells at the 4-cell stage in medaka embryos can differ in volumes over a large scale. The inequality of cell volume directly reflects the grade of asymmetric cell cleavage. In zebrafish it has been reported that the cell cycle becomes asynchronous at cleavage 9, and it was suggested that this asynchrony is due to volume differences that may result from unequal early cell divisions [2]. However, this study did not provide cell volume data prior to cycle 9. We speculate that unequal cell volumes at cycle 9 in zebrafish are just the consequence of early asymmetric cell divisions and early unequal cell volumes like it is the case for early medaka.

### Early RNA polymerase II transcriptional activity

Phosphorylation of the carboxy terminal domain (CTD) of RNA polymerase II (RNAPII) is associated with active RNA transcription [25] [26] [27] [28]. In medaka, phosphorylation was detected first during division from 8 to 16 cells, but phosphorylation stayed rare and sporadic until the 16-cell and 32-cell stages. Consistent phosphorylation of RNAPII in a large fraction of cells is established only by the 64-cell stage, even though cells at the periphery of the embryo usually do not show any phosphorylation until early blastula stage.

A lack of phosphorylation was noted in more centrally located cells of some embryos at the 128-cell stage. RNAPII is hypophosphorylated during mitosis [29] as transcription is repressed at this process [30]. In consequence, this could indicate that the cell cycle in embryos of this stage becomes metasynchronous as cells are at different steps of the cell cycle. This pattern is blurred in later stages, when phosphorylation-positive and negative cells are strongly mixed together and the phosphorylation pattern develops a more mosaic character. This change is caused by the increase in cell asynchrony during the embryos transition towards early blastula and the beginning MBT.

Importantly, until now only one study attempted to identify the time point of MBT activation in medaka by investigating the first appearances of paternal transcripts by RT-PCR. This study determined the beginning of MBT in medaka at around stage 11 (2000 to 4000cells) [6]. In contrast, our study proves that RNA polymerase II gets phosphorylated in a small fraction of cells already at the 16-/32-cell stages and strongly in larger fractions by the 64-cell stage. Furthermore, our findings also demonstrate that transcription in medaka embryos is detectable at earlier time points (between 64 to 1000 cells) than it was assumed so far. Previous microarray analysis on mRNA levels of over 16.000 genes from zebrafish embryos have shown that mRNA levels of 125 genes are increasing at the 64-cell stage. Many of these genes are involved in protein degradation, cell proliferation, cell growth, cell adhesion and RNA synthesis and stability. It was supposed that these genes need to commence their function during a pre-MBT state to ensure full functionality during MBT and following embryonic development.

The data reported here for early medaka embryo development conflict with the general descriptions for fish embryos before the midblastula transition, namely being synchronous, with equal cell division and no transcription occurring before MBT, which was set to stages beyond 512 cells.

Still, our results do not contradict the previously postulated mechanisms for MBT onset. Chromatin regulation, maternal clock, transcript abortion and nucleo-cytoplasmic ratio are all mechanistically compatible with our observations.

The here reported unequal distribution of cytoplasm, the wave-like cell cycle pattern together with very early RNAPII-activity and the initiation of transcription before MBT do, however, demonstrate that early development of medaka is more robust and less strictly controlled than was expected. Furthermore, our measurements imply that MBT is not an all-or-nothing, digitally-switched process, but rather a progressive event that is independently occurring in individual cells.

## Materials and Methods

### Ethic statement

This research did neither involve human participants nor any human material. All experiments involving live animals were conducted in accordance with the German Animal Protection Law. Our laboratory and animal facilities are approved for such work and are regularly inspected and supervised by the animal protection officers of the University of Würzburg and the Government of Lower Franconia. OLAW: A-5864-01.

### Fishes

Medaka fishes (*Oryzias latipes)* of the Carbio strain (Carolina Biological Supplies, USA) were kept as a large random-mating colony under standard conditions at a 14 h light-cycle and 24°C room temperature. Embryos were collected 20minutes after fertilization, extricated from the filament and raised in de-ionized water. Embryos where staged according to Iwamatsu [20].

### Light microscopy

Medaka embryos at the 4 cell stage were transferred into 2.5% methylcellulose and imaged using a M205FA Leica Microscope. Images were processed with ImageJ.

### Fluorescent staining and confocal microscopy

Embryos were cooled down to 4°C to slow down the cell cycle and then fixed in 4% PFA/1×PBS over night (o/n) at room temperature (RT). After fixation, embryos were rinsed in 1×PBS for 20″ at RT and stored in PBS at 4°C for 48 h. The chorion was physically removed using forceps and the embryos were detached from the yolk.

For cell cycle measurements, DNA was stained with Hoechst (Invitrogen (Hoechst 34580: H21486); Available: http://products.invitrogen.com/ivgn/product/H21486?ICID=search-product. Accessed 20 Jun 2011.) in a 1∶2000 dilution in PBS for 2″ and washed 2 times in PBS for 10″ and additionally in fresh PBS o/n at 4°C. The next day, embryos were transferred to 30 µl of fresh PBS on a microscope slide, covered with a cover slip and scanned immediately with a Nikon C1 confocal microscope.

For cell volume measurements, embryos were stained with the membrane dye CellMask DeepRed (Invitrogen (Available: CellMask DeepRed: C10046) http://products.invitrogen.com/ivgn/product/C10046. Accessed 20 Jun 2011) in a 1∶1000 dilution in PBS for 10″ and washed in o/n in PBS at 4°C. Right before washing, DNA was stained by adding Hoechst in a 1∶2000 dilution for 2″. After washing, embryos were transferred into DABCO over night and were fixed in Mowiol on a microscope slide on the next day. Confocal images were taken using a Leica TCS SP5 confocal microscope.

For p-polymerase II detection, embryos were fixed in PFA, stored in PBS at 4°C, removed from the yolk as described above. Embryos were permeabilised with 0.1% TritonX in PBS for 10″, blocked in 5% BSA in PBS for 1–2 h and incubated with anti p-RNAPII antibody (SantaCruz (p-Pol II (8A7): sc-13583); Available: http://www.scbt.com/datasheet-13583-p-pol-ii-8a7-antibody.html. Accessed 20 Jun 2011) at 1∶1000 for 48 h at 4°C. After first antibody incubation, a 10″ washing in PBS for 4 times at RT followed. Detection was done with an Alexa568-coupled anti mouse antibody at 1∶1000 in 5%BSA for 24 h at 4°C. DNA was stained with Hoechst. Confocal imaging was done with a Nikon C1 confocal microscope.

### Cell cycle analysis

Cell cycle synchrony was investigated in previously DNA stained and confocal imaged embryos. For visualization, confocal stacks were loaded into the Volocity V5.3.2 imaging software (PerkinElmer) and filtered.

Embryos were classified as asynchronous if cells at different stages of the cell cycle (Interphase/Mitotic phase) were found within the embryo. Cells were considered as being in interphase as long as only 1 nuclear signal within the same cell was detected. The splitting into two individual nuclear signals was considered as breaking point for classifying the cell as being in mitotic phase. This is accompanied by an elongated shape and symmetry along an imaginary plane between both chromosome-sets. Loss of the elongated shape and the clearly detectable symmetry of the nuclear signal were considered to mark the end of the mitotic phase and the re-entry into interphase.

Embryos were also classified as asynchronous if all cells indeed showed mitotic character, but are at different time points or progression levels of the metaphase. A close position between the daughter chromosomes represents nuclei that have just entered anaphase, whereas a large distance represents nuclei that are at late ana- or telophase and have entered mitosis prior to the early anaphase cells.

### Cell volume determination

Confocal images were loaded into the Volocity software and filtered. Single cells were isolated by manually performed optical dissection on individual embryos. Only embryos that did not change the shape and X-/Y-position of their cleavage furrow along the Y-axis were used for this procedure. Cell volumes were measured in Volocity with an object-identifying protocol that was adjusted at the beginning and remained unchanged for all cell volume measurements. The volumes of all 4 cell volumes of each embryo were summed up and set as 100%, and the relative volume of each cell was calculated relative to the total volume of the particular embryo. These relative volumes were used for comparisons of cell volumes within individual embryos and between different embryos. Embryos were considered as “consisting of cells with similar volumes“ if the relative volumes'differences between the smallest and the largest cell did not exceed 5%, which is a volume difference between both cells close to or smaller than the factor 1.2 from the smallest to the largest cell. Embryos were considered as “consisting of three cells of similar volume and an extremely large or small fourth cell” if the differences between the relative volumes'differences of the smallest and the largest cells to the embryos mean relative volume did not exceed the factor 2. The embryos mean relative volume was calculated between the 2^nd^ smallest and the 2^nd^ largest cell: {[(B+C)/2]-A}/{D-[(B+C)/2]} with A as the smallest and D as the biggest cell. Additional information for the calculations can be found in Table S1.

### In vitro mRNA transcription and mRNA injection and confocal imaging

Histone2B-eGFP mRNA was transcribed with the mMESSAGE mMACHINE SP6-Kit (Ambion). The template for in-vitro transcription was obtained via PCR from the pCS2P-plasmids backbone in which the Histone2B-eGFP sequence was cloned. Primers 5′-ATTTAGGTGACACTATAG-3′ and 3′-CAGGAAACAGCTATGACCATGATTACG-5′ were used for template amplification. Medaka eggs were injected at the late stage 0. For imaging, embryos were mounted in 0.7% low melting point agarose in H_2_O on a coverslip that was glued into the hole of a microscope slide. The eggs were orientated with the animal pole facing down and subsequently scanned with a Nikon C1 confocal microscope with a 4.107 minute delay between each stack.

Nuclear size was determined as the area measured by the contrast based “Find 2D Nuclei” measurement protocol in the Volocity software on maximum intensity projections of confocal stacks.

### Expression analysis and RT-PCR

Total RNA of medaka embryos at different stages was isolated as previously described [31]. RNA amounts for reverse transcription were measured with the Qubit-Kit (Invitrogen). Investigated stages were stage 0–2 (1–4 cells), stage 8–10 (64–1000 cells), stage 11 and stage 24. Quantitative real-time PCR was performed with SYBR Green reagents in a reaplex^2^ Mastercycler (Eppendorf). Target genes were orthologs of zebrafish genes for which up-regulation in zebrafish embryos after the 64-cell stage had been reported [7]. Used primer pairs were intron-spanning and are listed in Table S2. All results are averages of three independent experiments. Error bars represent standard deviation of the mean. Relative expression levels were calculated after correction to ef1a1 expression, which was used as housekeeping gene.

### Statistical analysis

Significance values for cell division desynchronization and upcoming metasynchronous cell division, for asymmetry compensation, for symmetry/metasynchrony correlation and for asymmetry/cell-volume correlation were calculated using the Chi-square test. Significance values for RT-PCR were calculated with the Student' t-test. Significance values for polymerase II phosphorylation was calculated with the Welch' t-test.

A probability of p<0,05 was considered as statistically significant; * and ** and *** represent statistical significances below p<0.05, p<0.01 and p<0.001 respectively.

## Supporting Information

Figure S1 **Hoechst staining at cell division from 128 to 256 cells.** (A–C) Different time points (early-late) during cell division from 128 cells to 256 cells. (A) Interphase. All cells are in interphase (yellow arrows). (B) Early phase. Cell division starts first in central cells (green arrows), while peripheral cells do not yet divide (yellow arrows). (C) Late phase. Central cells have finished mitosis (yellow arrows) when peripheral cells undergo mitosis (green arrows).
(TIF)

Figure S2 **Cell divisions at mid-late cleavage phases of a symmetric dividing embryo after H2B-eGFP-injection.** Cell division progression of an embryo that has divided symmetrically from 2 to 4 cells at 4 successive cell cycles. (A) Embryo at three different time points (interphase, early-, late- phase) at cycle 6, 32 to 64 cells. Early cell division appears in random positioned cells of the embryo at early phase (green arrows). Other cells have not entered interphase (yellow arrows). (B–D) Embryo at three different time points (interphase, early-, late- phase) at cycle 7 (64 to 128 cells), cycle 8 (128 to 256 cells) and cycle 9 (256–512 cells), respectively. Cell division starts first in central positioned cells (green arrows) during early phase of the embryós cycle. Peripheral cells have not yet entered cell division (yellow arrows). Central positioned cells have started to enter interphase (yellow arrows) and peripheral cells are still in ana-/telophase of the cell cycle at later phase of the embryós cycle.
(TIF)

Figure S3 **Nucleus size progression during cleavage phase.** Progression of nuclear sizes (as an area measurement) in a mid-interphase cell at the 8 cell stage and in its derived daughter cells until to the late 64 cell stage. The nuclear area was determined every 4.107 min for each nucleus for 45 consecutive time points. Nuclear areas are large at mid-interphase cells, small before cell division and smallest after cell division. No detectable desynchronization until measurement point 27 (red circles). Early mitosis in cells at more central position (blue circle). Chromosomes are condensed in late cells, but not separated (red arrows). Nuclear size is shown on the Y-axis (in µm^2^), measuring points at the X-axis. Overall temporal progression is shown at the bottom. Arrows lead from a single cell to the according daughter cells after each cell cycle.
(TIF)

Figure S4 **Potential for a clear metasynchronous division pattern after dividing symmetrically or asymmetrically from 2 to 4 cells.** H2B-eGFP mRNA injected embryos are shown that divided symmetrically and asymmetrically from 2 to 4 cells. Embryos that divided symmetrically more often (37/40 with 92.5%) developed a clear metasynchronous division pattern than asymmetrically divided embryos (18/39 with 46,2%) (values are given in percentages; Chi-square test with p<0,001).
(TIF)

Figure S5 **Cell divisions at mid-late cleavage phases of an asymmetric dividing embryo after H2B-eGFP-injection.** Cell division progression of two embryos that have divided asymmetrically from 2 to 4 cells at 2 successive cell cycles. (A–B) Embryo at three different time points (interphase, early-, late- phase) at cycle 7 (64 to 128 cells) and 8 (128 to 256 cells) are shown. Cell division is early in cells that are positioned on the left side of the embryo during early phase of the embryós cycle (green arrows). Cell cycle is late in cells that are located on the right side of the embryo (yellow arrows). During the late phase of the cell cycle division is finished in cells that are located on the left side (yellow arrows) but is still undergo in cells on the right side of the embryo (green arrows). (C–D) Embryo at three different time points (interphase, early-, late-phase) at cycle 7 (64 to 128 cells) and 8 (128 to 256 cells) are shown. Cell division is early in cells that are positioned on the left side of the embryo during early phase of the embryós cycle (green arrows). Cell cycle is late in cells that are located on the right side of the embryo (yellow arrows). During the late phase of the cycle cell division is finished in cells that are located on the left side (yellow arrows) but is still undergo in cells on the right side of the embryo (green arrows).
(TIF)

Figure S6 **Examples for type III embryos from medaka fish.** (A–F) Different examples for type III embryos from medaka fish. Cell borders are highlighted.
(TIF)

Figure S7 **Individual interphase stages during cleavage phase of a type I embryo.** (A–I) Developmental stages of a type I medaka embryo from the 4-cell stage to the 1024-cell stage are shown.
(TIF)

Figure S8 **Compensation disadvantages after asymmetric cleavage from 2 to 4 cells.** Figure illustrates the potential to compensate the asymmetric cell divisions in type II and type III embryos. Type I embryos (green bars) were used as positive control as they usually develop like the ideal Iwamatsu embryo. Type II (blue bars) or type III embryos (orange bars) that could no longer be distinguished from type I embryos were counted as an ideal embryo. At the 4-cell stage, 100% of the type I embryos were counted as ideal and none of the type II or type III embryos. At the 128-cell stage, the number of ideal type I embryos dropped as 5% of this embryo fraction no longer could be counted as ideal as the remaining type I embryos. The number of ideal type II and type III embryos rose to 86% (p<0.001) and 64% (p<0.001) respectively. At the 1000-cell stage, all type I and all type II embryos showed the ideal shape and the number of ideal type III also increased to 91% (p<0.030). (Chi-square test).
(TIF)

Figure S9 **Individual interphase stages during cleavage phase of a type III embryo.** (A–I) Developmental stages of a type III medaka embryo from the 4-cell stage to the 1024-cell stage are shown.
(TIF)

Figure S10 **Cell shape changes in 3D.** Confocal scans of medaka embryos at the 4-cell stage stained with Orange CellMask. (A–F; G–L) Images of 2 different embryos at 3 different positions (near top, middle, near bottom) on the z-axis are shown. (D and F; J and L) Cell borders of top and bottom position are highlighted. (E; K) Overlay of top and bottom borders are merged.
(TIF)

Figure S11 **Correlation between asymmetric cell divisions and cell volume differences.** Illustration of the dispersion of the three embryo types among the investigated embryos regarding the cell size differences between the largest and the smallest cell of each embryo. Bars show the frequency of each embryo type among the embryo-fraction representing the 50% embryos with the smallest differences (embryos 1–16) and the 50% embryos with the largest differences (embryos 17–33). Type I embryos are represented by green bars, type II by blue bars and type III embryos by orange bars. Type I embryos show a similar distribution between the fractions of the 16 most similar and most dissimilar embryos like the type II embryos (Chi-square test with p = 0.3382). Type III embryos instead are more associated with the dissimilar fraction than type I embryos (p<0.001) and type II embryos (p = 0.00135).
(TIF)

Figure S12 **Cell volumes at the 4-cell stage regarding the embryo types I-III.** Cell volumes of medaka embryos at the 4-cell stage are illustrated regarding the different embryo types I (A), type II (B), type III (C). Embryos containing cells with small differences are oriented to the left. Differences are increasing to the right.
(TIF)

Figure S13 **Fold changes in cell volume at the 4-cell stage regarding the embryo types I-III.** Fold differences between the cell volume of the largest and the smallest cell within medaka embryos at the 4-cell stage are illustrated regarding the different embryo types type I (A), type II (B) and type III (C). Embryos containing cells with small fold change differences are oriented to the left.
(TIF)

Figure S14 **RNA Polymerase II phosphorylation in early embryos.** RNA Polymerase II phosphorylation in early medaka embryos at the 8-cell stage (A), the 512-cell stage (B), 1024-cell stage (C). No phosphorylation is detectable in cells at the 8-cell stage (A). Phosphorylation is prevalent in embryos at 512 cells (B) and at 1024 cells (C).
(TIF)

Figure S15 **Increase of polymerase II phosphorylation in early stages.** Levels of RNA polymerase II phosphorylation between the 8-cell and the 128-cell stage are shown. Values are given as percentages of all cells at the embryo stage to allow comparisons between each stage. No phosphorylated Pol II was detected before the 16-cell stage. At the 16-cell stage, p-pol II levels increase slightly to about 17% of to cells being positive (p = 0.002) and again to the 32-cell stage with a further slight increase to 30.5% positive cells (p = 0.0024). By reaching the 64-cell stage, p-pol II levels show a major increase to about 73% (p<0.001) and remain high at the 128-cell stage with 67.7% of all cells being positive (p = 0.1186). (Welch's t-test, error bars are standard deviations).
(TIF)

Table S1 **Cell volumes at the 4-cell stage and processed volume calculations.**
(XLSX)

Table S2 **RT-PCR primer list.**
(TIF)

Movie S1 **Bright field time lapse of normal medaka development.** Movie shows a developing medaka embryo between the 4-cell and 256-cell stage with a gap of 25 seconds between images.
(AVI)

Movie S2 **Confocal time lapse of normal medaka development after H2B-eGFP mRNA injection.** Movie shows a developing medaka embryo that has divided symmetrically to the 4 cell stage. Movie ranges from 4 cells to 1000–2000 cells with a gap of 4.107 minutes between images. The embryo develops a clear metasynchronous cell division.
(AVI)

Movie S3 **Confocal time lapse of asymmetric medaka development after H2B-eGFP mRNA injection.** Movie shows a developing medaka embryo that has divided asymmetrically to the 4 cell stage. Movie ranges from 4 cells to 1000–2000 cells with a gap of 4.107 minutes between images. The embryo is unable to develop a metasynchronous cell division but divides in waves that move from one pole of the embryo to the other pole.
(AVI)
