# Supplementary figures and images for: Highly Asynchronous and Asymmetric Cleavage Divisions Accompany Early Transcriptional Activity in Pre-Blastula Medaka Embryos
Source: PLoS One. 2011 Jul 7;6(7):e21741. doi: 10.1371/journal.pone.0021741 (PMC3131289; doi:10.1371/journal.pone.0021741)

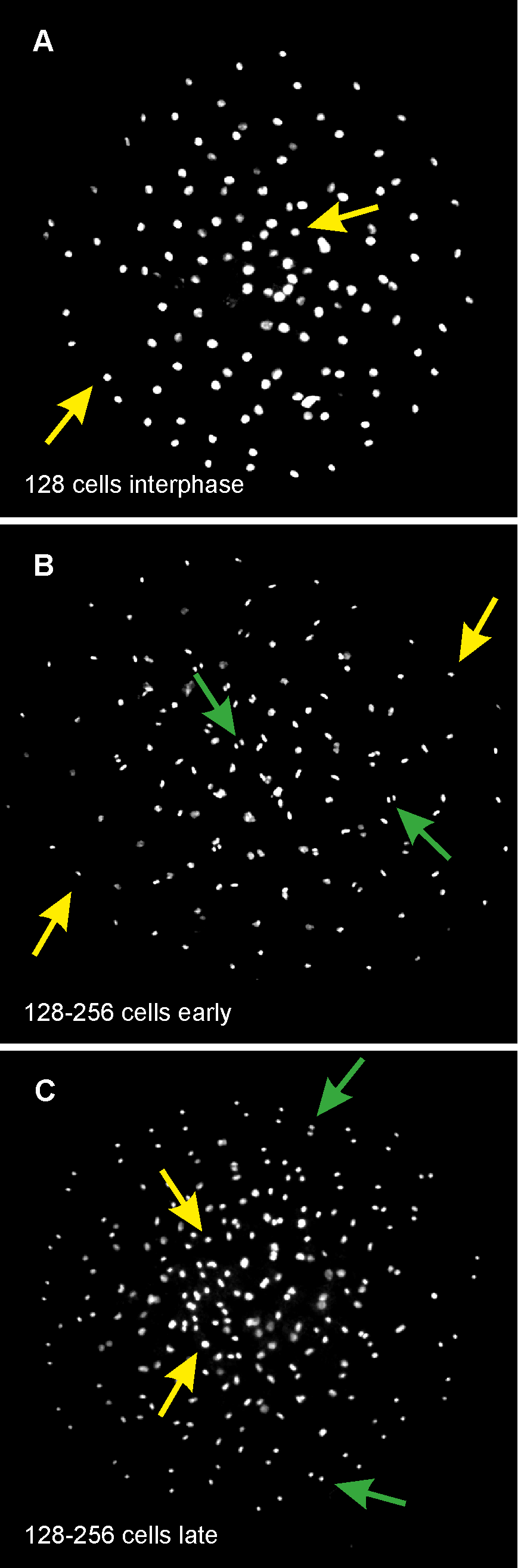

Supplement: Figure S1 — Hoechst staining at cell division from 128 to 256 cells. (A–C) Different time points (early-late) during cell division from 128 cells to 256 cells. (A) Interphase. All cells are in interphase (yellow arrows). (B) Early phase. Cell division starts first in central cells (green arrows), while peripheral cells do not yet divide (yellow arrows). (C) Late phase. Central cells have finished mitosis (yellow arrows) when peripheral cells undergo mitosis (green arrows). (TIF) [file pone.0021741.s001.tif]

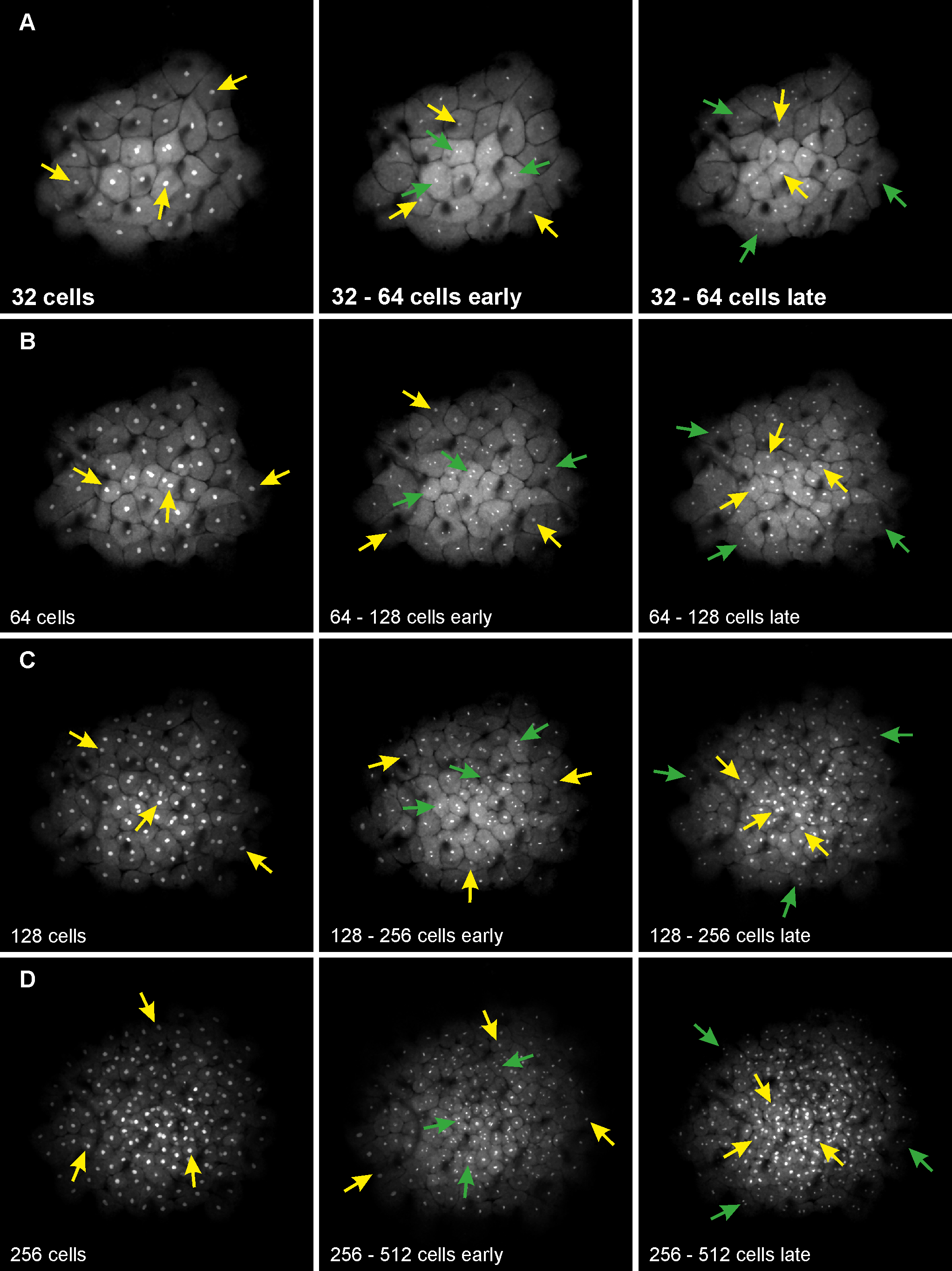

Supplement: Figure S2 — Cell divisions at mid-late cleavage phases of a symmetric dividing embryo after H2B-eGFP-injection. Cell division progression of an embryo that has divided symmetrically from 2 to 4 cells at 4 successive cell cycles. (A) Embryo at three different time points (interphase, early-, late- phase) at cycle 6, 32 to 64 cells. Early cell division appears in random positioned cells of the embryo at early phase (green arrows). Other cells have not entered interphase (yellow arrows). (B–D) Embryo at three different time points (interphase, early-, late- phase) at cycle 7 (64 to 128 cells), cycle 8 (128 to 256 cells) and cycle 9 (256–512 cells), respectively. Cell division starts first in central positioned cells (green arrows) during early phase of the embryós cycle. Peripheral cells have not yet entered cell division (yellow arrows). Central positioned cells have started to enter interphase (yellow arrows) and peripheral cells are still in ana-/telophase of the cell cycle at later phase of the embryós cycle. (TIF) [file pone.0021741.s002.tif]

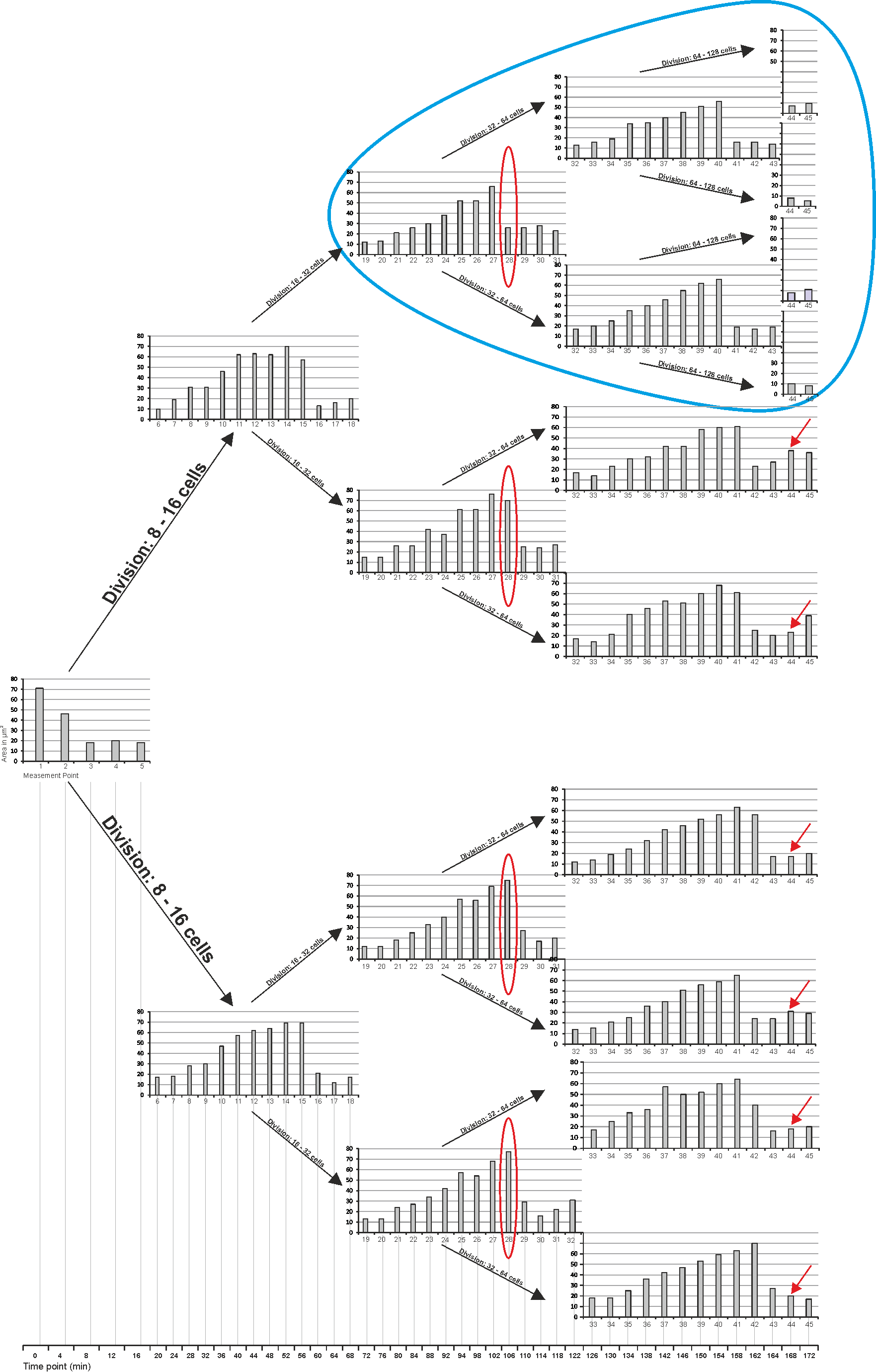

Supplement: Figure S3 — Nucleus size progression during cleavage phase. Progression of nuclear sizes (as an area measurement) in a mid-interphase cell at the 8 cell stage and in its derived daughter cells until to the late 64 cell stage. The nuclear area was determined every 4.107 min for each nucleus for 45 consecutive time points. Nuclear areas are large at mid-interphase cells, small before cell division and smallest after cell division. No detectable desynchronization until measurement point 27 (red circles). Early mitosis in cells at more central position (blue circle). Chromosomes are condensed in late cells, but not separated (red arrows). Nuclear size is shown on the Y-axis (in µm2), measuring points at the X-axis. Overall temporal progression is shown at the bottom. Arrows lead from a single cell to the according daughter cells after each cell cycle. (TIF) [file pone.0021741.s003.tif]

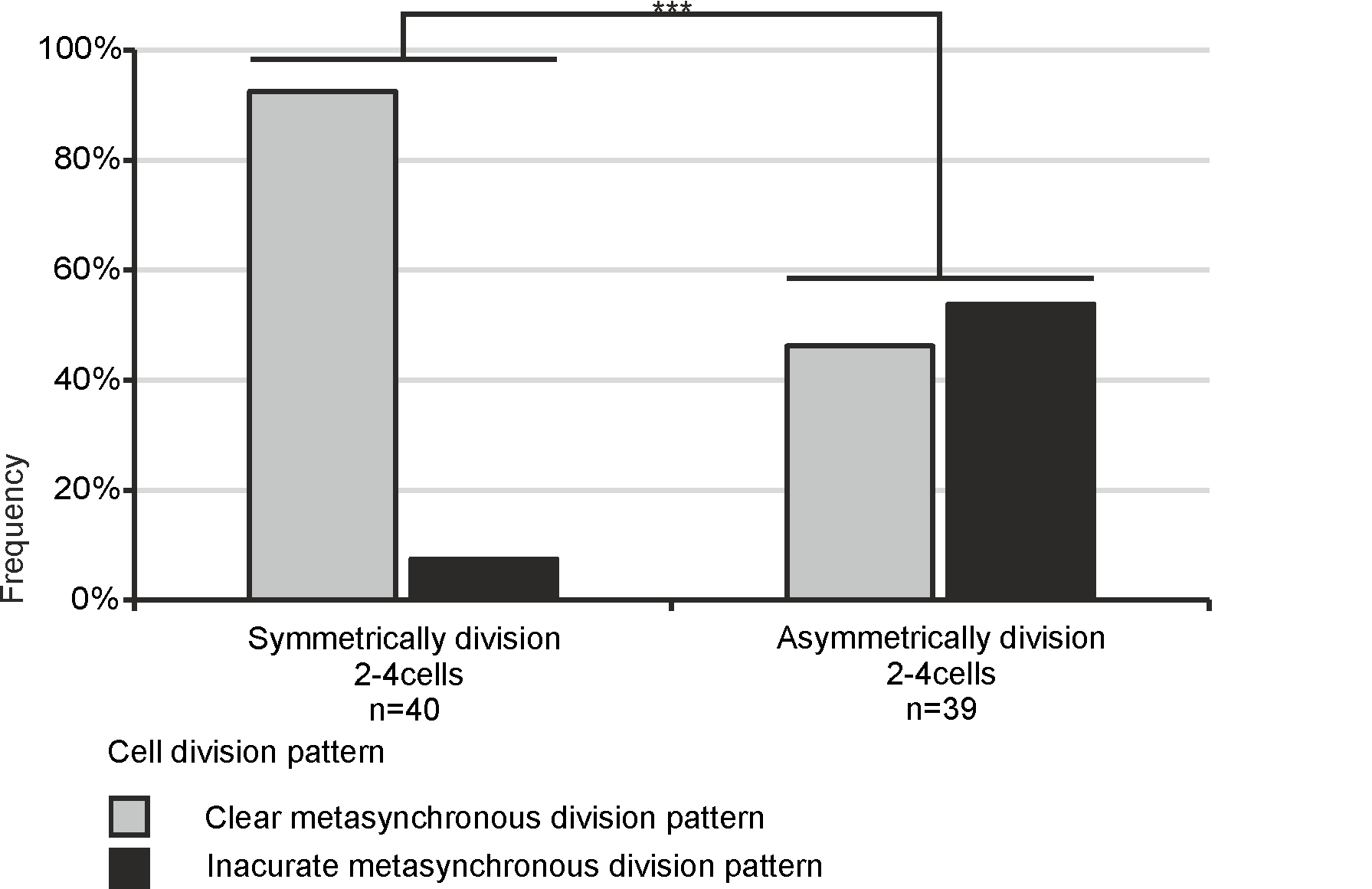

Supplement: Figure S4 — Potential for a clear metasynchronous division pattern after dividing symmetrically or asymmetrically from 2 to 4 cells. H2B-eGFP mRNA injected embryos are shown that divided symmetrically and asymmetrically from 2 to 4 cells. Embryos that divided symmetrically more often (37/40 with 92.5%) developed a clear metasynchronous division pattern than asymmetrically divided embryos (18/39 with 46,2%) (values are given in percentages; Chi-square test with p<0,001). (TIF) [file pone.0021741.s004.tif]

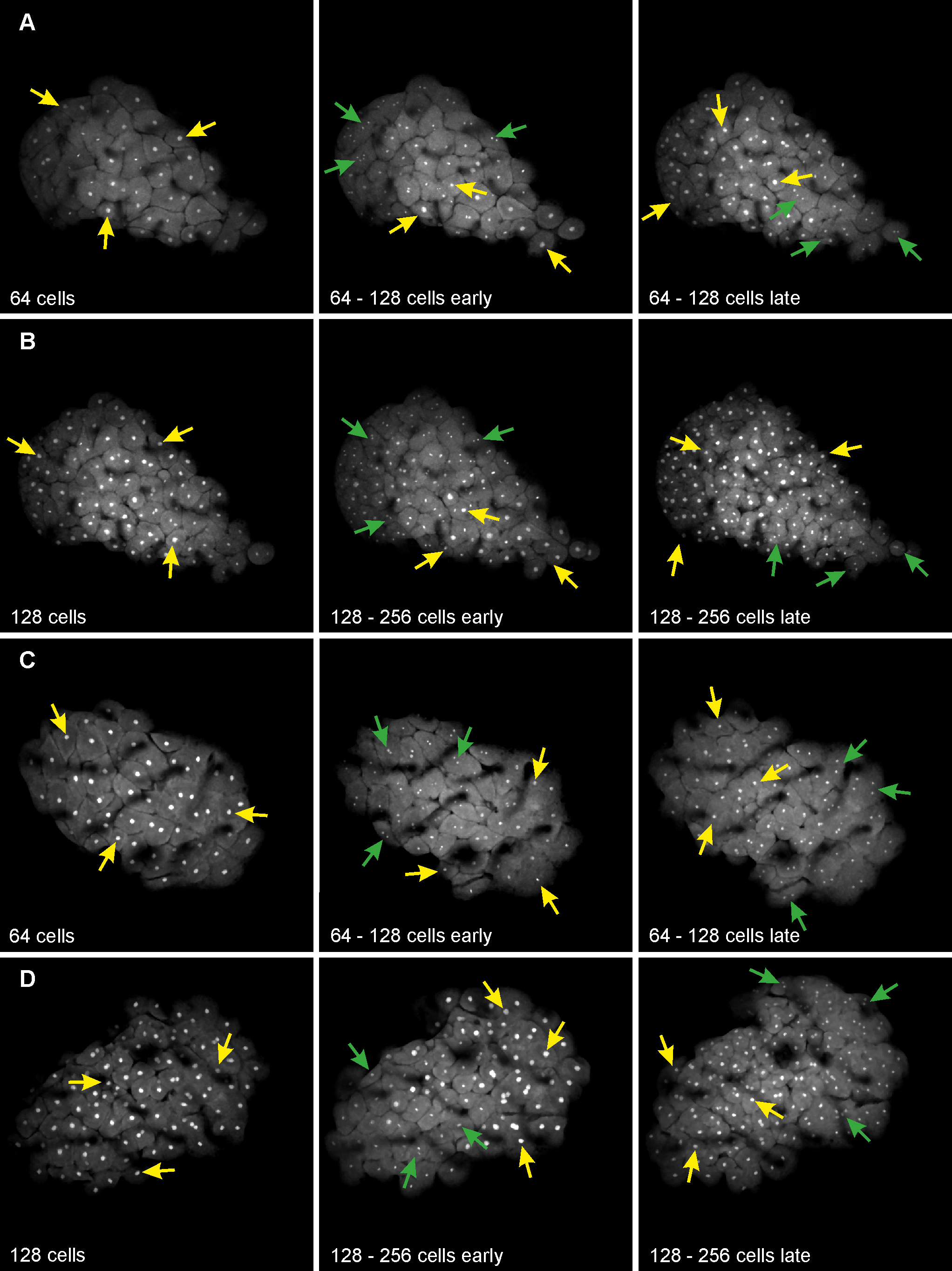

Supplement: Figure S5 — Cell divisions at mid-late cleavage phases of an asymmetric dividing embryo after H2B-eGFP-injection. Cell division progression of two embryos that have divided asymmetrically from 2 to 4 cells at 2 successive cell cycles. (A–B) Embryo at three different time points (interphase, early-, late- phase) at cycle 7 (64 to 128 cells) and 8 (128 to 256 cells) are shown. Cell division is early in cells that are positioned on the left side of the embryo during early phase of the embryós cycle (green arrows). Cell cycle is late in cells that are located on the right side of the embryo (yellow arrows). During the late phase of the cell cycle division is finished in cells that are located on the left side (yellow arrows) but is still undergo in cells on the right side of the embryo (green arrows). (C–D) Embryo at three different time points (interphase, early-, late-phase) at cycle 7 (64 to 128 cells) and 8 (128 to 256 cells) are shown. Cell division is early in cells that are positioned on the left side of the embryo during early phase of the embryós cycle (green arrows). Cell cycle is late in cells that are located on the right side of the embryo (yellow arrows). During the late phase of the cycle cell division is finished in cells that are located on the left side (yellow arrows) but is still undergo in cells on the right side of the embryo (green arrows). (TIF) [file pone.0021741.s005.tif]

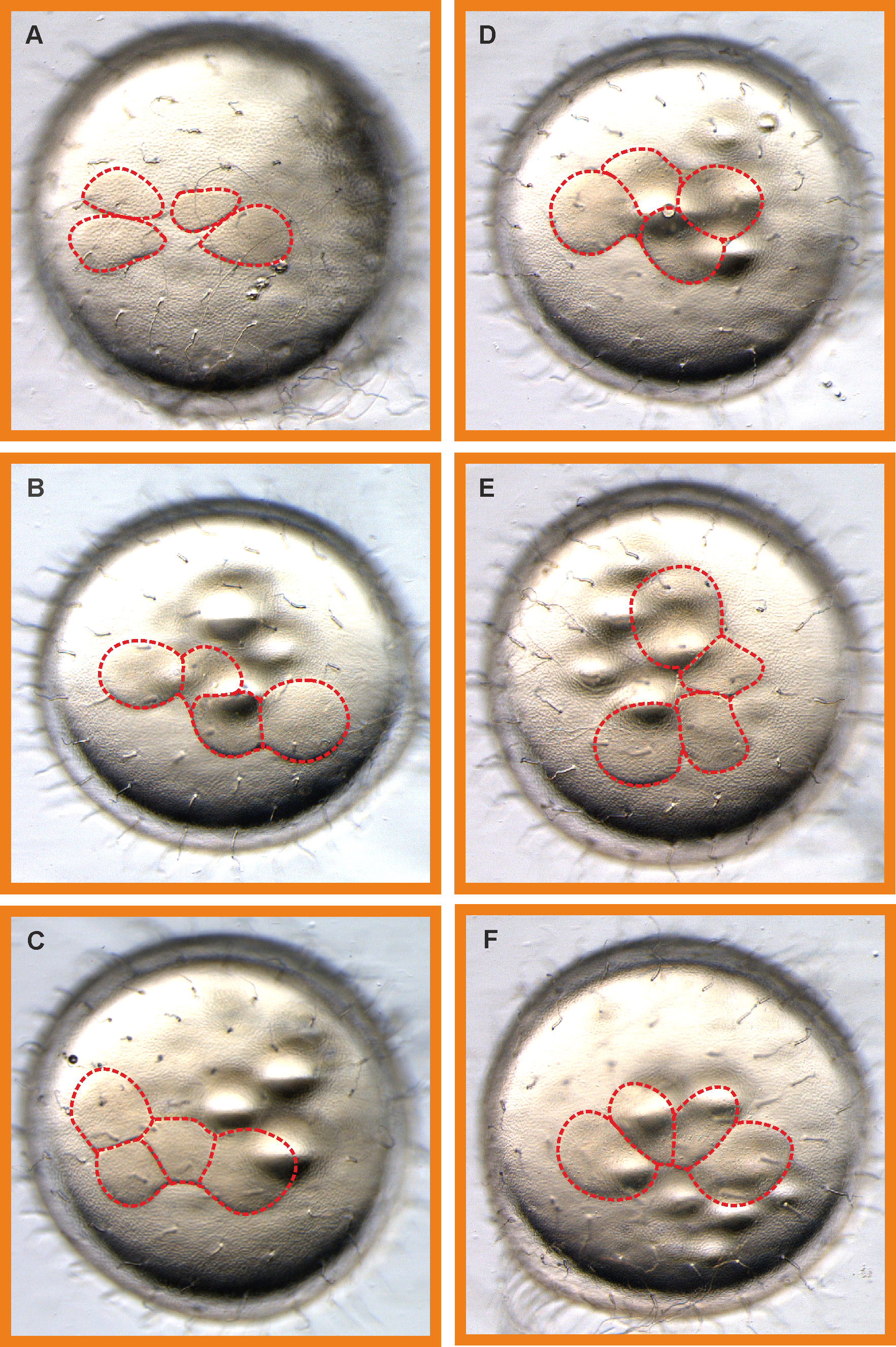

Supplement: Figure S6 — Examples for type III embryos from medaka fish. (A–F) Different examples for type III embryos from medaka fish. Cell borders are highlighted. (TIF) [file pone.0021741.s006.tif]

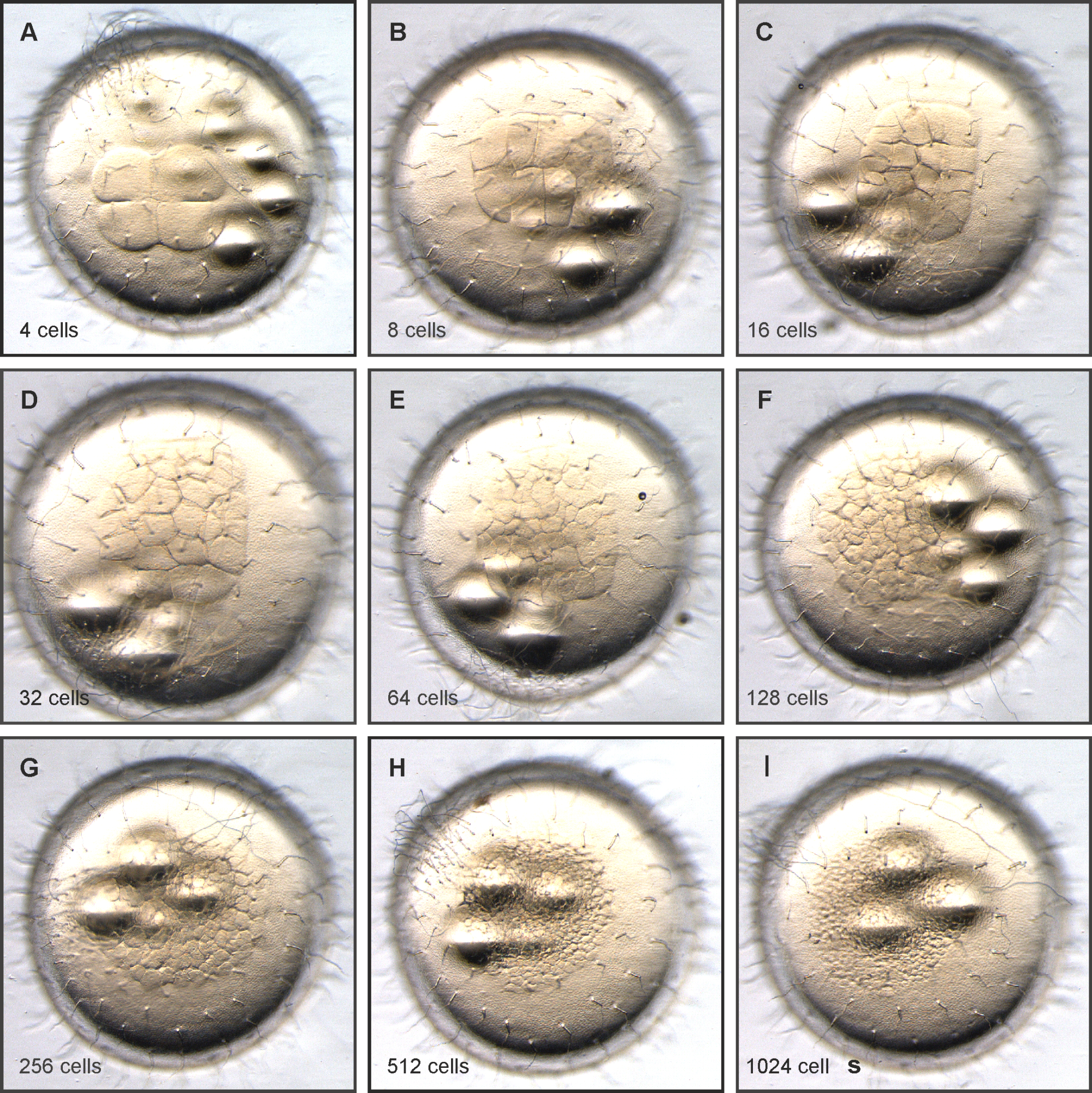

Supplement: Figure S7 — Individual interphase stages during cleavage phase of a type I embryo. (A–I) Developmental stages of a type I medaka embryo from the 4-cell stage to the 1024-cell stage are shown. (TIF) [file pone.0021741.s007.tif]

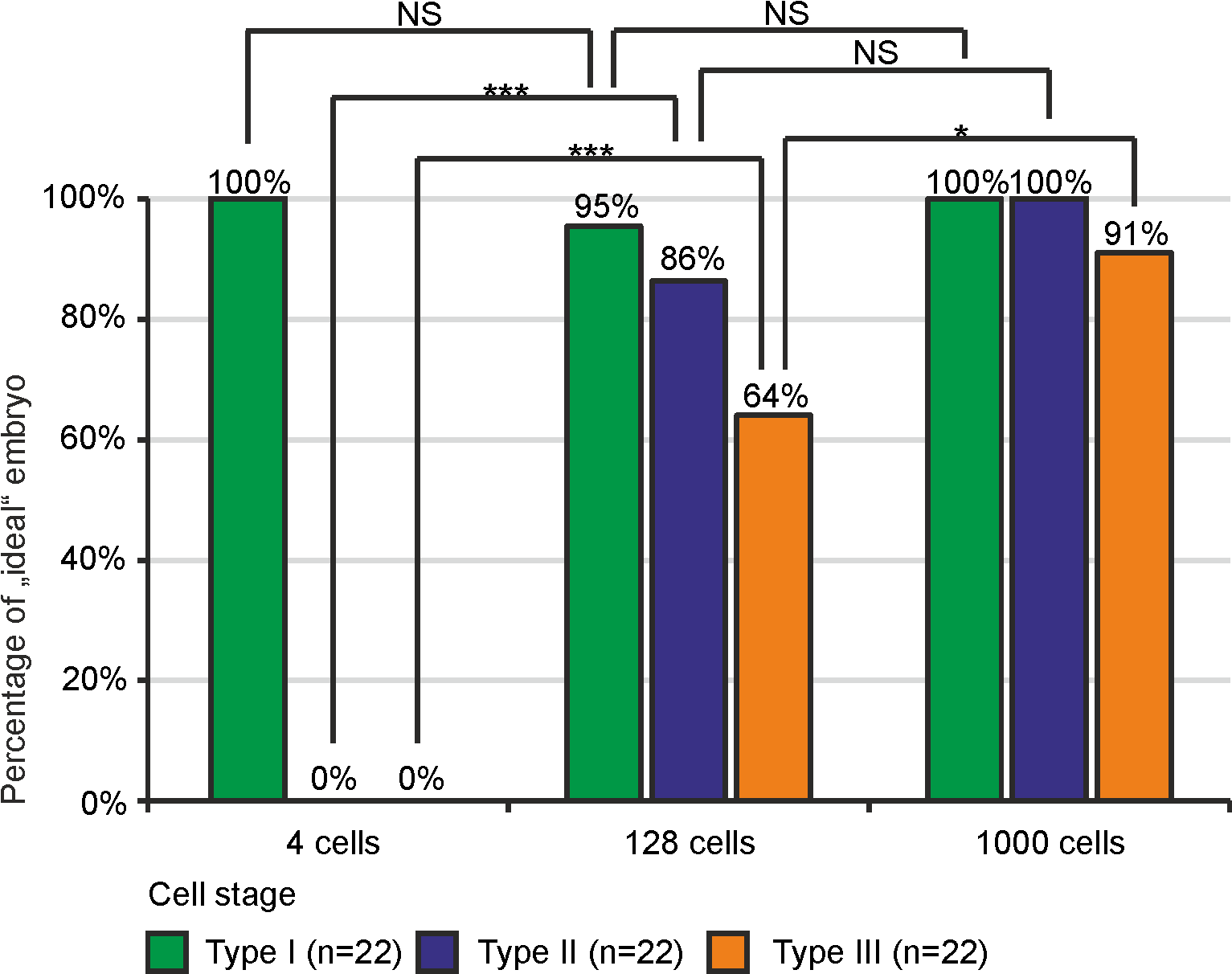

Supplement: Figure S8 — Compensation disadvantages after asymmetric cleavage from 2 to 4 cells. Figure illustrates the potential to compensate the asymmetric cell divisions in type II and type III embryos. Type I embryos (green bars) were used as positive control as they usually develop like the ideal Iwamatsu embryo. Type II (blue bars) or type III embryos (orange bars) that could no longer be distinguished from type I embryos were counted as an ideal embryo. At the 4-cell stage, 100% of the type I embryos were counted as ideal and none of the type II or type III embryos. At the 128-cell stage, the number of ideal type I embryos dropped as 5% of this embryo fraction no longer could be counted as ideal as the remaining type I embryos. The number of ideal type II and type III embryos rose to 86% (p<0.001) and 64% (p<0.001) respectively. At the 1000-cell stage, all type I and all type II embryos showed the ideal shape and the number of ideal type III also increased to 91% (p<0.030). (Chi-square test). (TIF) [file pone.0021741.s008.tif]

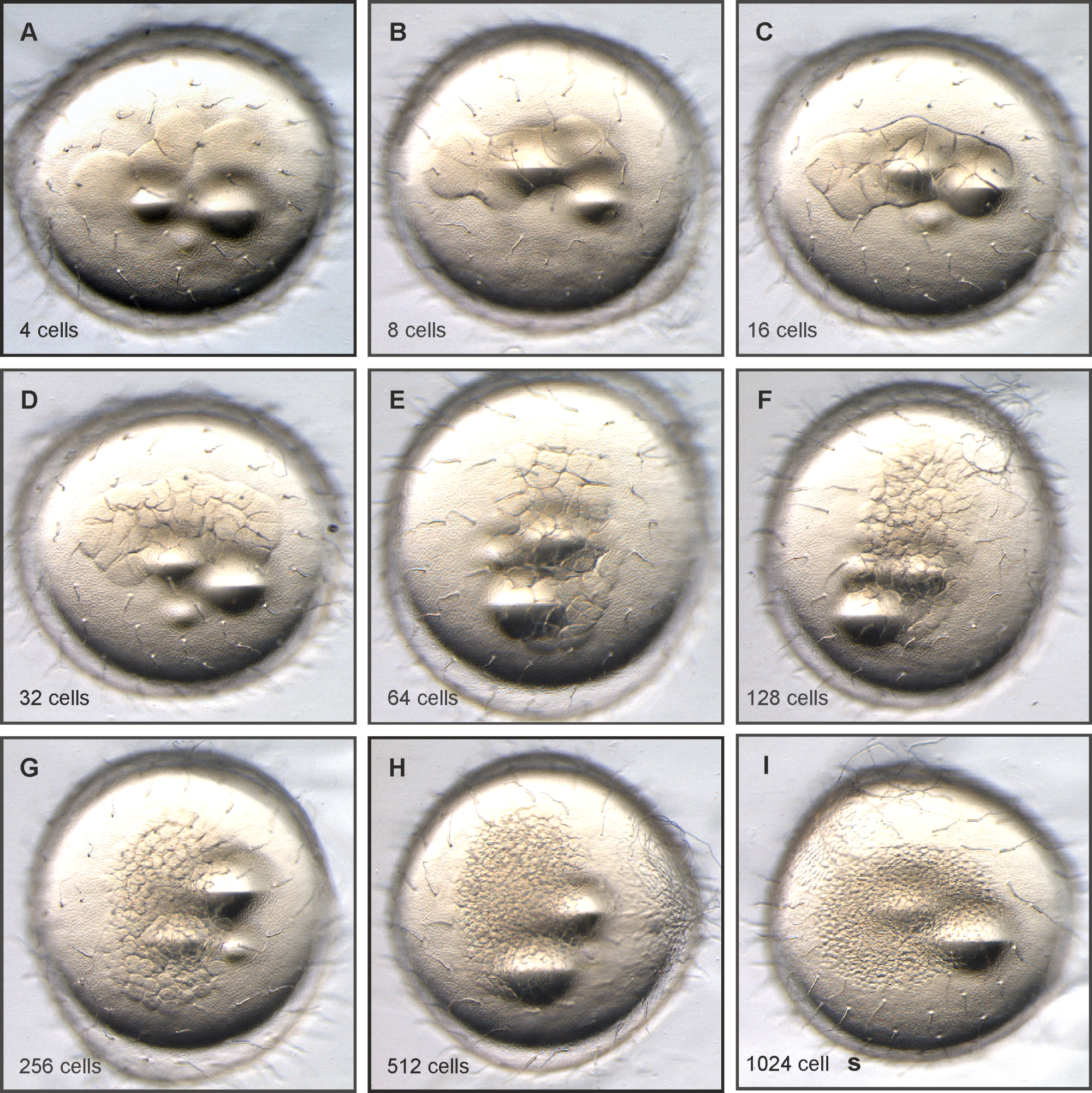

Supplement: Figure S9 — Individual interphase stages during cleavage phase of a type III embryo. (A–I) Developmental stages of a type III medaka embryo from the 4-cell stage to the 1024-cell stage are shown. (TIF) [file pone.0021741.s009.tif]

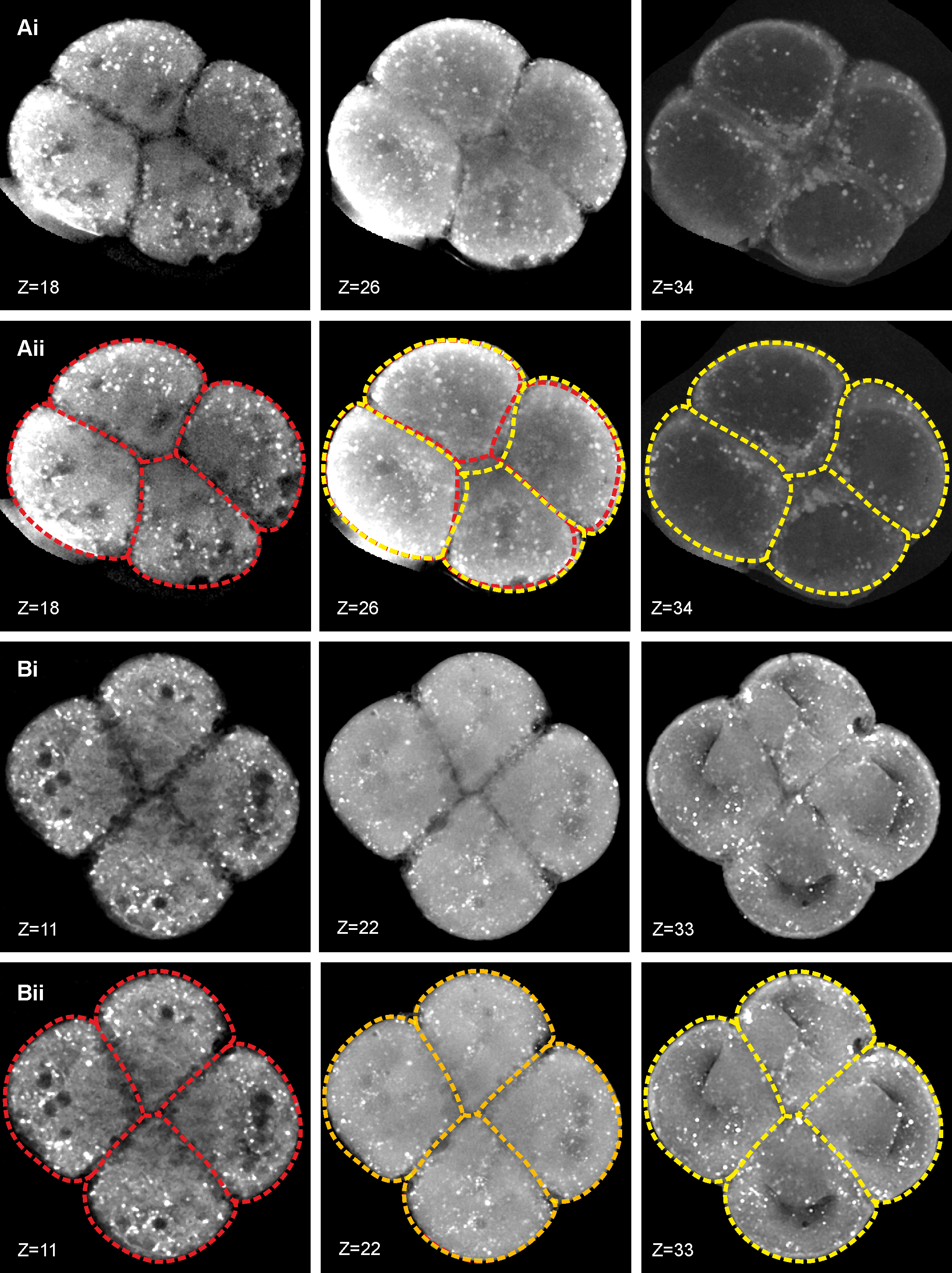

Supplement: Figure S10 — Cell shape changes in 3D. Confocal scans of medaka embryos at the 4-cell stage stained with Orange CellMask. (A–F; G–L) Images of 2 different embryos at 3 different positions (near top, middle, near bottom) on the z-axis are shown. (D and F; J and L) Cell borders of top and bottom position are highlighted. (E; K) Overlay of top and bottom borders are merged. (TIF) [file pone.0021741.s010.tif]

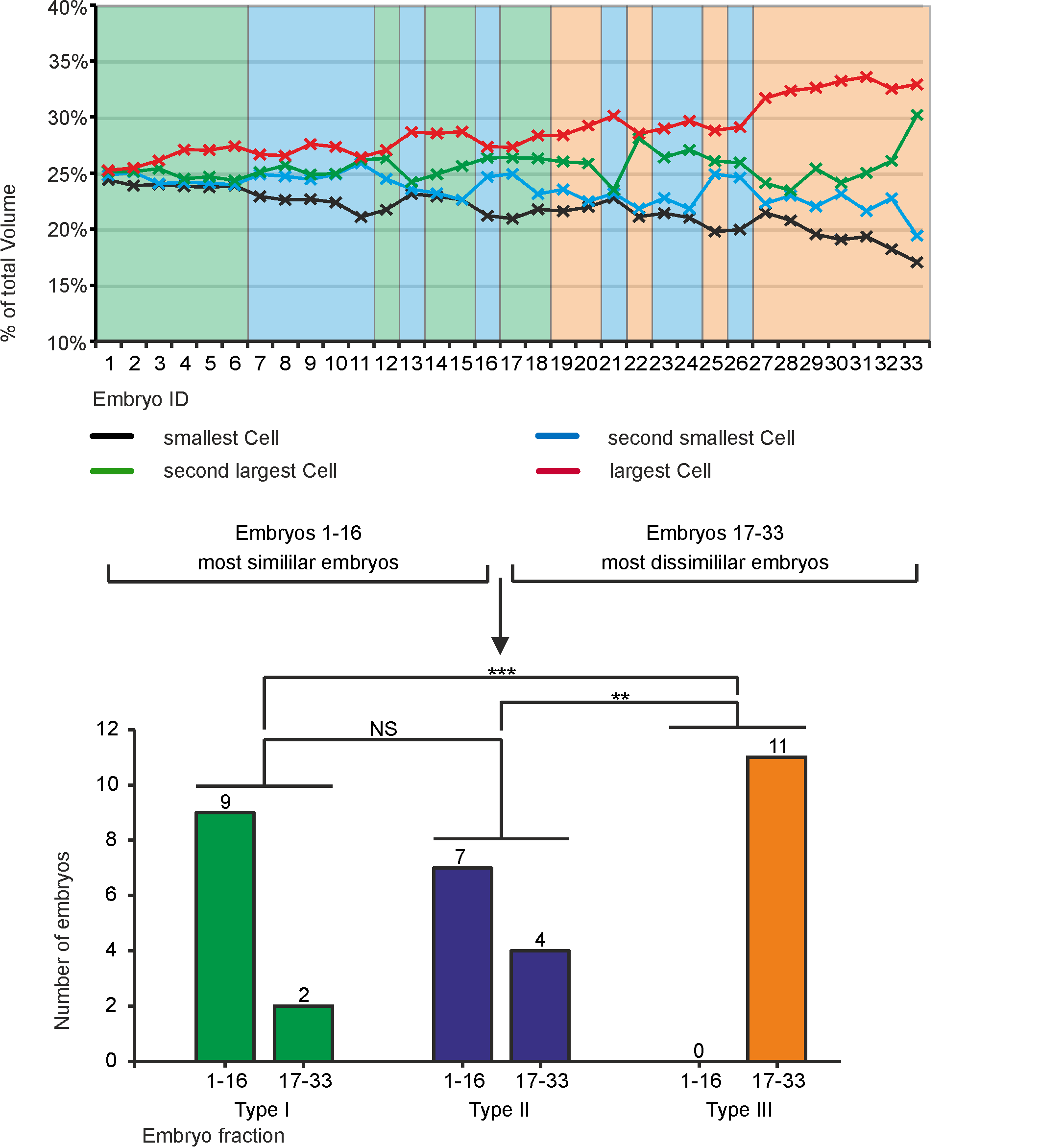

Supplement: Figure S11 — Correlation between asymmetric cell divisions and cell volume differences. Illustration of the dispersion of the three embryo types among the investigated embryos regarding the cell size differences between the largest and the smallest cell of each embryo. Bars show the frequency of each embryo type among the embryo-fraction representing the 50% embryos with the smallest differences (embryos 1–16) and the 50% embryos with the largest differences (embryos 17–33). Type I embryos are represented by green bars, type II by blue bars and type III embryos by orange bars. Type I embryos show a similar distribution between the fractions of the 16 most similar and most dissimilar embryos like the type II embryos (Chi-square test with p = 0.3382). Type III embryos instead are more associated with the dissimilar fraction than type I embryos (p<0.001) and type II embryos (p = 0.00135). (TIF) [file pone.0021741.s011.tif]

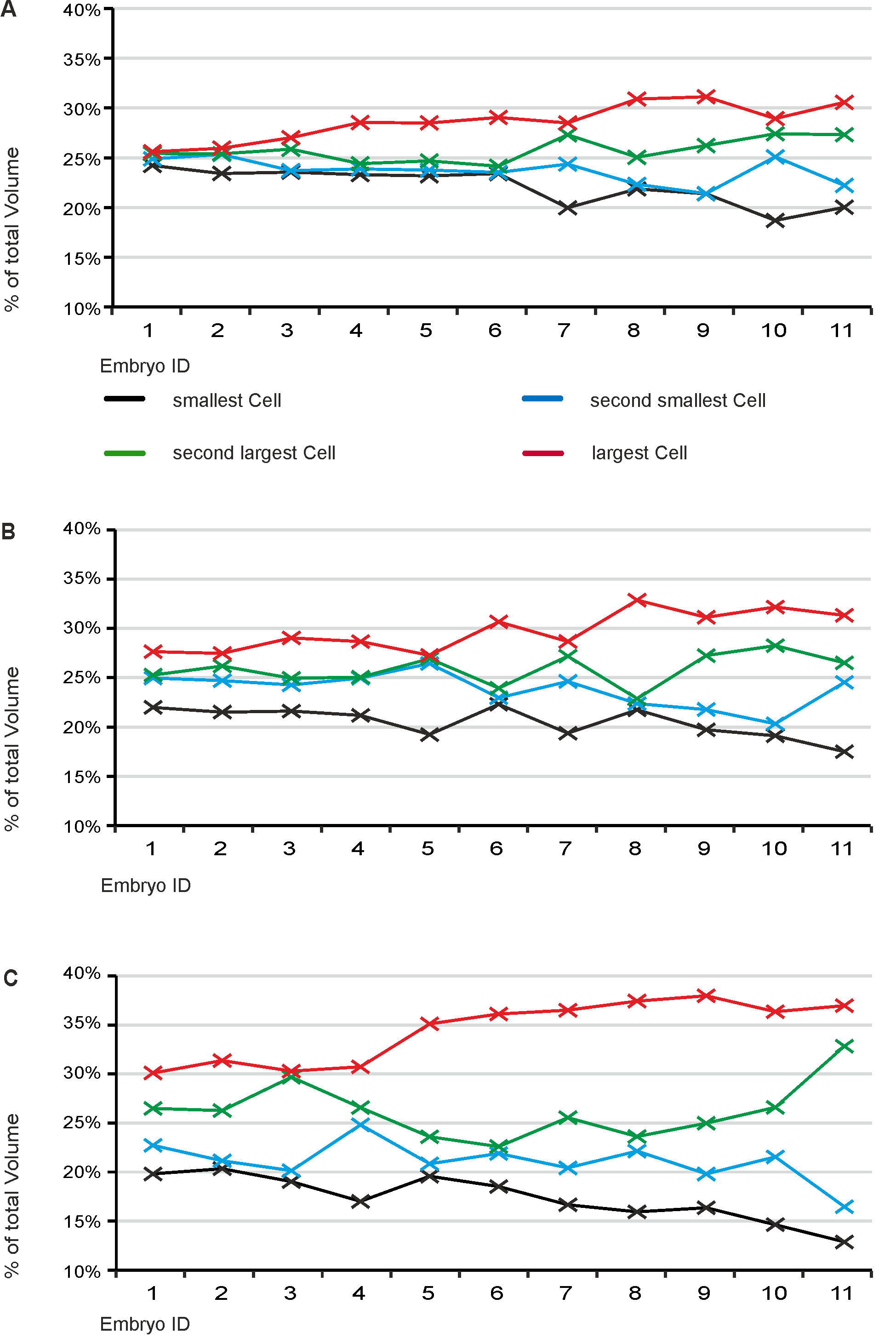

Supplement: Figure S12 — Cell volumes at the 4-cell stage regarding the embryo types I-III. Cell volumes of medaka embryos at the 4-cell stage are illustrated regarding the different embryo types I (A), type II (B), type III (C). Embryos containing cells with small differences are oriented to the left. Differences are increasing to the right. (TIF) [file pone.0021741.s012.tif]

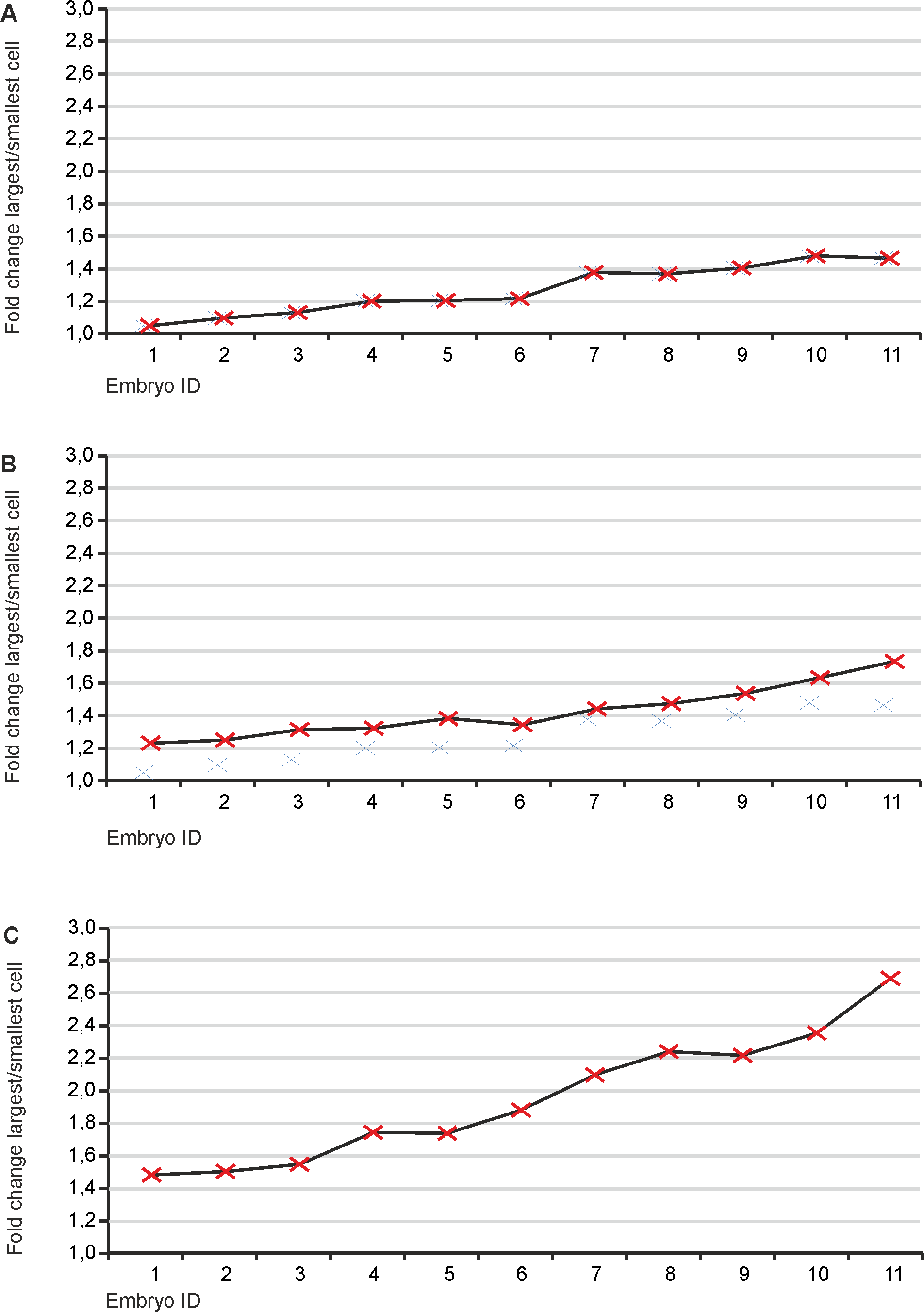

Supplement: Figure S13 — Fold changes in cell volume at the 4-cell stage regarding the embryo types I-III. Fold differences between the cell volume of the largest and the smallest cell within medaka embryos at the 4-cell stage are illustrated regarding the different embryo types type I (A), type II (B) and type III (C). Embryos containing cells with small fold change differences are oriented to the left. (TIF) [file pone.0021741.s013.tif]

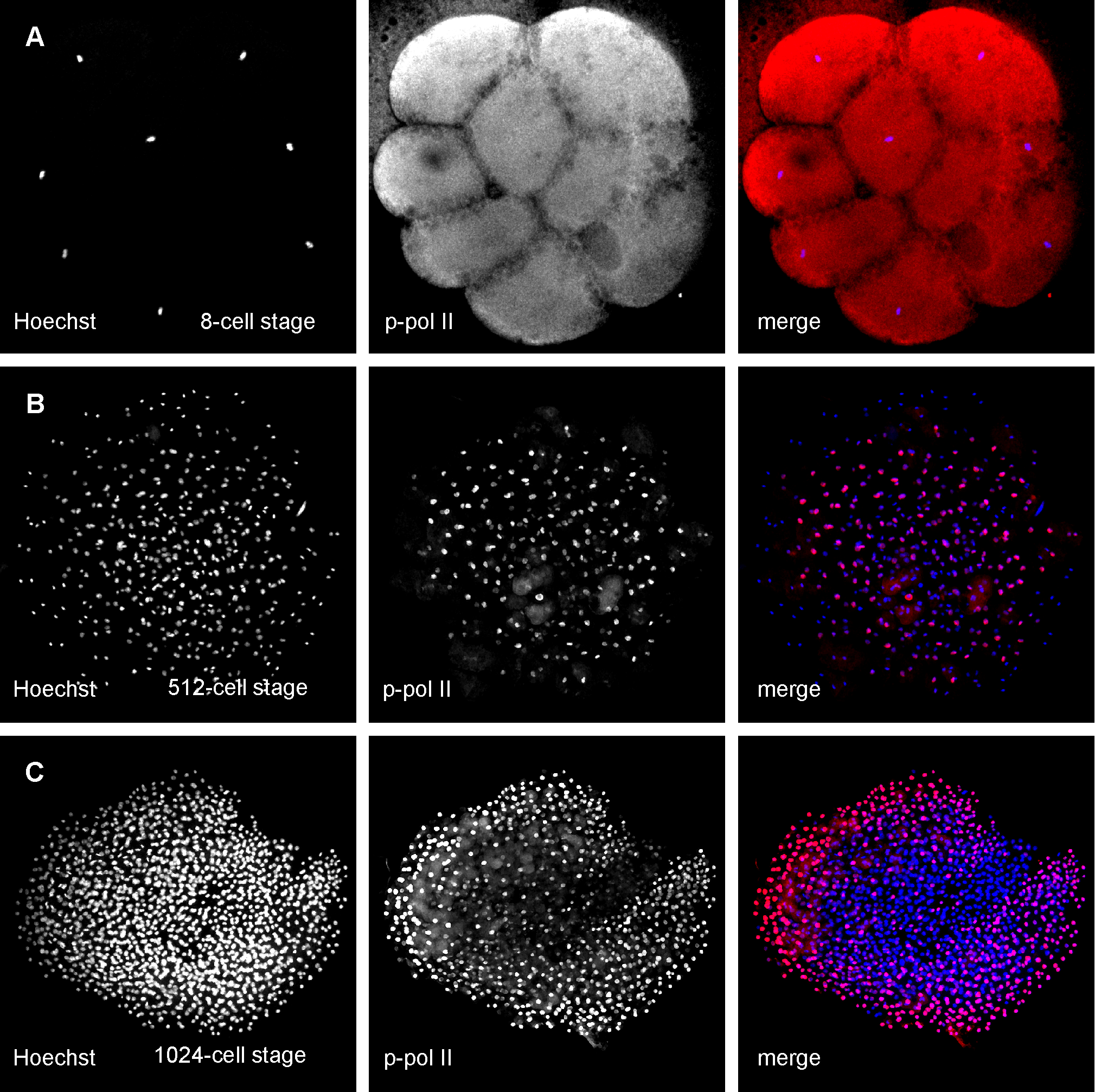

Supplement: Figure S14 — RNA Polymerase II phosphorylation in early embryos. RNA Polymerase II phosphorylation in early medaka embryos at the 8-cell stage (A), the 512-cell stage (B), 1024-cell stage (C). No phosphorylation is detectable in cells at the 8-cell stage (A). Phosphorylation is prevalent in embryos at 512 cells (B) and at 1024 cells (C). (TIF) [file pone.0021741.s014.tif]

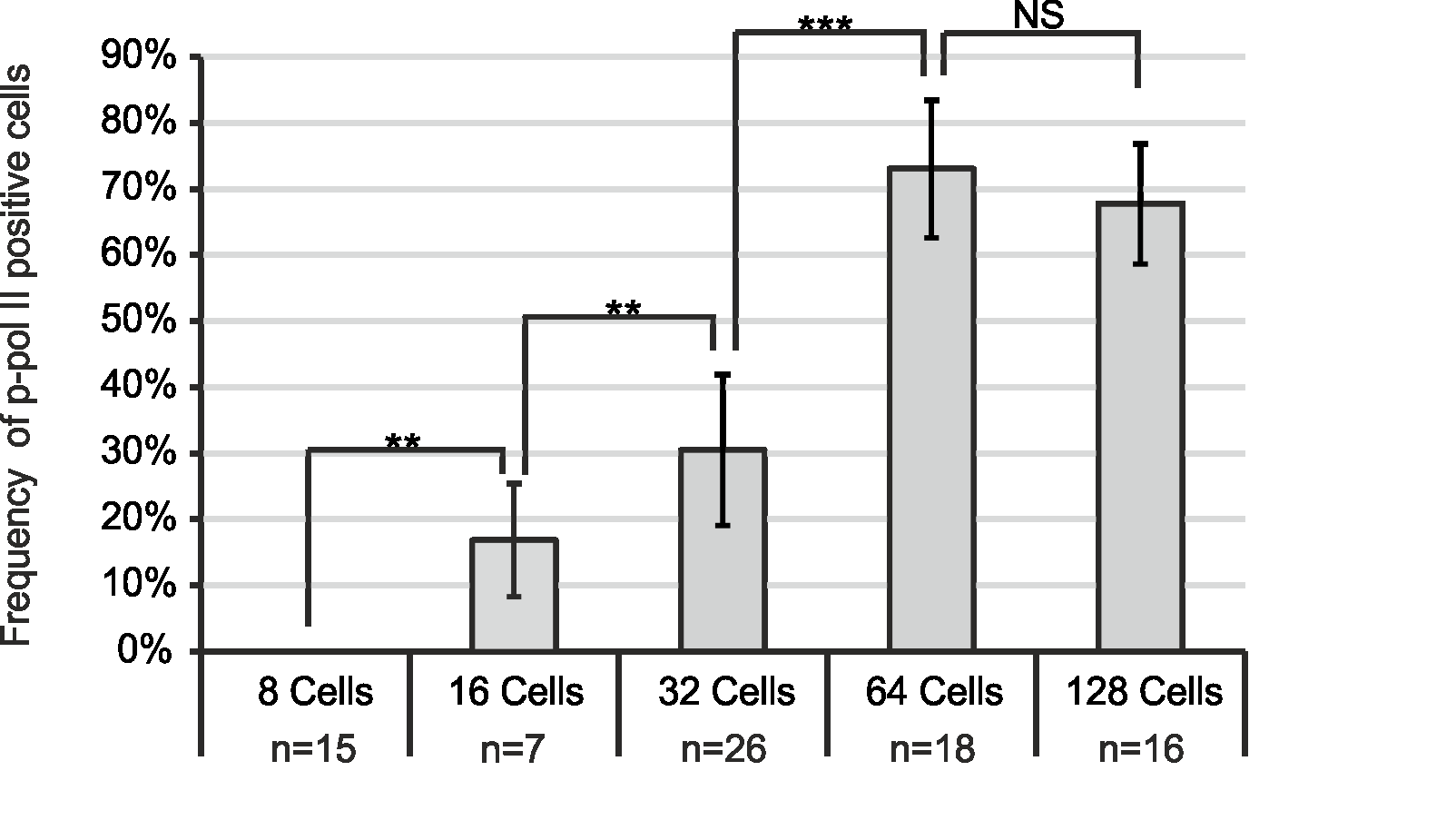

Supplement: Figure S15 — Increase of polymerase II phosphorylation in early stages. Levels of RNA polymerase II phosphorylation between the 8-cell and the 128-cell stage are shown. Values are given as percentages of all cells at the embryo stage to allow comparisons between each stage. No phosphorylated Pol II was detected before the 16-cell stage. At the 16-cell stage, p-pol II levels increase slightly to about 17% of to cells being positive (p = 0.002) and again to the 32-cell stage with a further slight increase to 30.5% positive cells (p = 0.0024). By reaching the 64-cell stage, p-pol II levels show a major increase to about 73% (p<0.001) and remain high at the 128-cell stage with 67.7% of all cells being positive (p = 0.1186). (Welch's t-test, error bars are standard deviations). (TIF) [file pone.0021741.s015.tif]

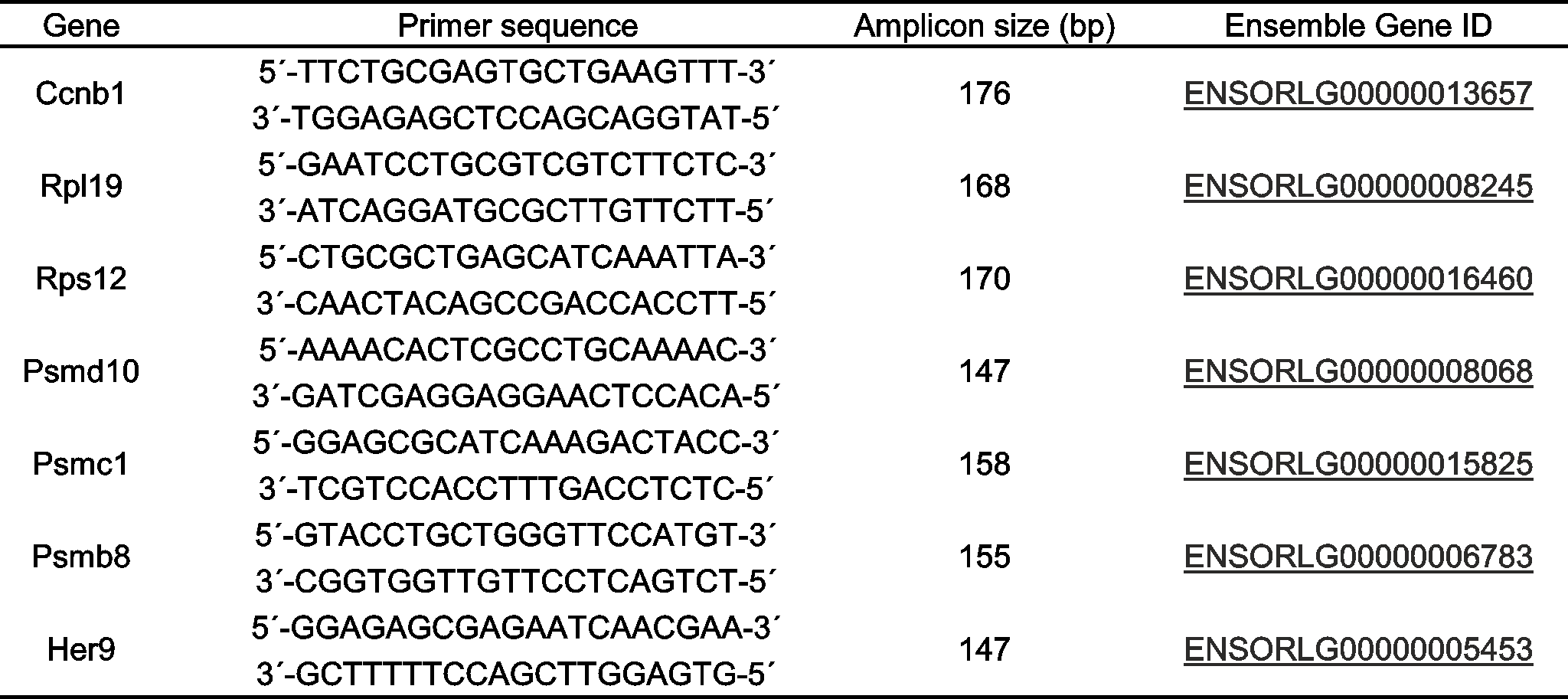

Supplement: Table S2 — RT-PCR primer list. (TIF) [file pone.0021741.s017.tif]
